# Mitochondrial dysfunction in neurodegenerative disorders

**DOI:** 10.1016/j.neurot.2023.10.002

**Published:** 2023-12-19

**Authors:** Madelyn M. Klemmensen, Seth H. Borrowman, Colin Pearce, Benjamin Pyles, Bharatendu Chandra

**Affiliations:** a University of Iowa Roy J and Lucille A Carver College of Medicine, Iowa City, IA 52242, USA; b Division of Medical Genetics and Genomics, Stead Family Department of Pediatrics, University of Iowa Hospitals and Clinics, Iowa City, IA 52242, USA; c Aper Funis Research, Union River Innovation Center, Ellsworth, ME 04605, USA

**Keywords:** Mitochondria, Neurodegeneration, Bioenergetics, Aging, Reactive oxygen species (ROS)

## Abstract

Recent advances in understanding the role of mitochondrial dysfunction in neurodegenerative diseases have expanded the opportunities for neurotherapeutics targeting mitochondria to alleviate symptoms and slow disease progression. In this review, we offer a historical account of advances in mitochondrial biology and neurodegenerative disease. Additionally, we summarize current knowledge of the normal physiology of mitochondria and the pathogenesis of mitochondrial dysfunction, the role of mitochondrial dysfunction in neurodegenerative disease, current therapeutics and recent therapeutic advances, as well as future directions for neurotherapeutics targeting mitochondrial function. A focus is placed on reactive oxygen species and their role in the disruption of telomeres and their effects on the epigenome. The effects of mitochondrial dysfunction in the etiology and progression of Alzheimer's disease, amyotrophic lateral sclerosis, Parkinson's disease, and Huntington's disease are discussed in depth. Current clinical trials for mitochondria-targeting neurotherapeutics are discussed.

## Graphical abstract

Overview/graphical abstract. Above is an overview figure highlighting common causes of mitochondrial dysfunction, proteinopathies and buildup, and associated outcomes. The graphical abstract was created using Biorender.

**Figure:**
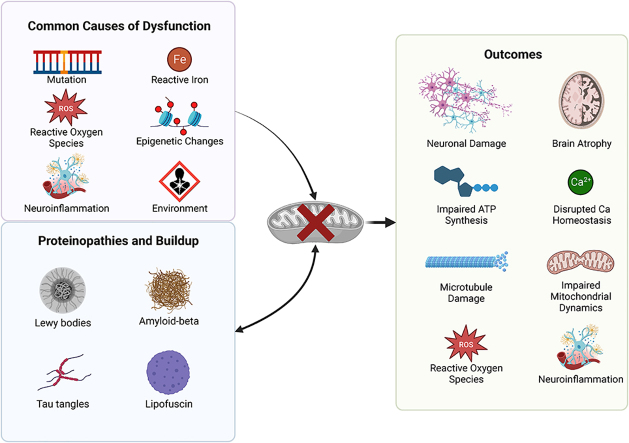

## Centrality of mitochondria in neurodegeneration

Mitochondria hold a central position in the biology of cells and are crucial to life. Eukaryotic cells contain many mitochondria, which occupy as much as a quarter of the cytoplasmic volume [1]. The secrets of the mitochondria's functions were not revealed until the 1950s, when Palade and Sjostand discovered the mitochondria's complex internal structure in electron microscopy studies. Due to the importance of mitochondria in producing energy for cells, life ultimately depends on their proper functioning. Mitochondrial dysfunction can lead to a variety of neurodegenerative disorders. Neurological and neuromuscular syndromes are the most frequent clinical presentations of mitochondrial disorders [2]. Although the density of mitochondria may be lower in neurons than it is in other cells, such as myocytes, the brain consumes almost ten times more oxygen and glucose compared to other tissues [3]. A resting cortical neuron consumes 4.7 billion ATP molecules per second 4], highlighting the dependence of the nervous system on mitochondria for its proper functioning. Mitochondria get transported across the neuron by miniature motors along tubular tracks to produce ATP where it's needed most. The highest demand for ATP in a neuron is usually in synapses, although some remain in basic housekeeping locations [4].

**Fig. 1:**
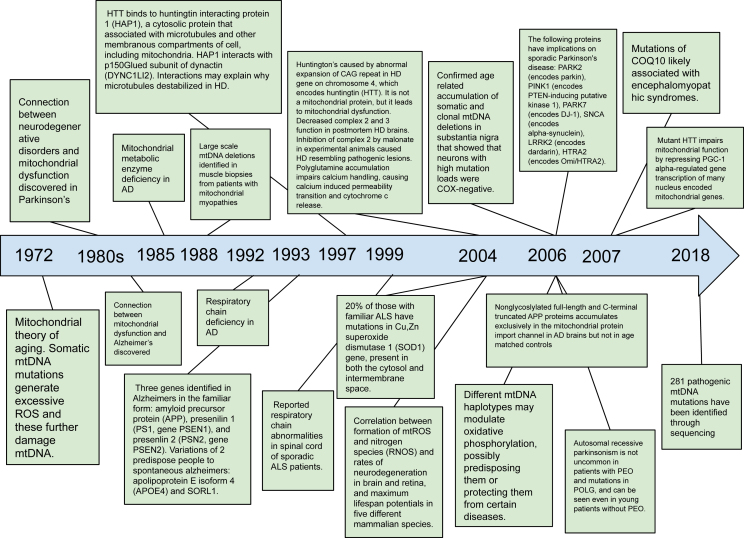
Timeline. Above is a historical timeline outlining seminal discoveries linking mitochondria to neurodegenerative disorders.

**Fig. 2:**
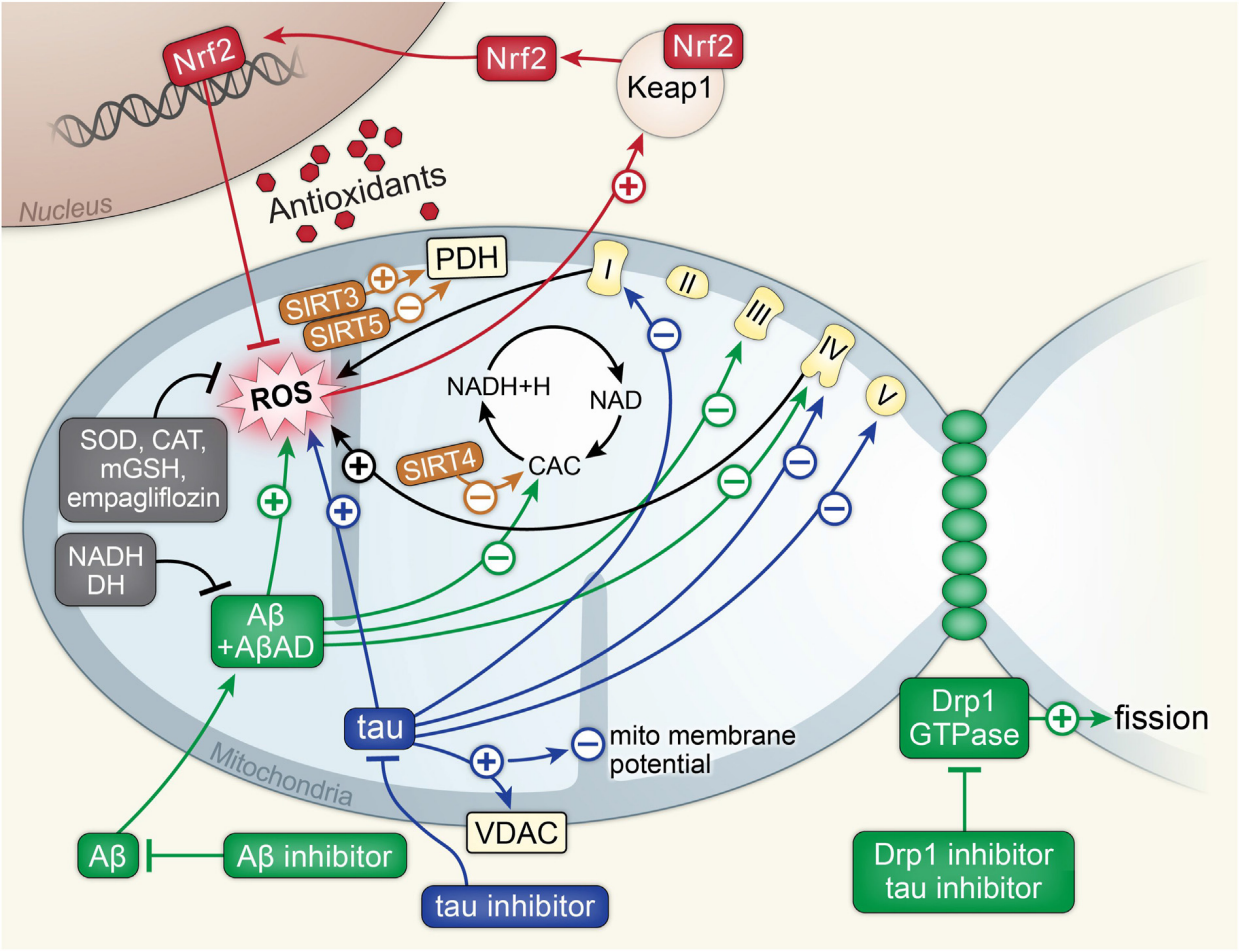
Alzheimer's disease. Above is a figure illustrating mitochondrial affecting pathways in Alzheimer's disease associated with Aβ, tau, Drp1, SIRT proteins, and Nrf2. Aβ and AβAD work synergistically to increase mitochondrial ROS, inhibit the CAC, and inhibit complexes 3 and 4 of the ETC. Aβ inhibitors inhibit Aβ and inhibit this synergistic activity. NADH and DH inhibit Aβ and AβAD synergy as well. Tau protein increases mitochondrial ROS and inhibits complexes 1, 4, and 5 of the ETC. Tau also increases the activity of VDAC, leading to the loss of the mitochondrial membrane potential. Tau inhibitors inhibit the tau protein. Drp1 works synergistically with GTPase to increase mitochondrial fission. Drp1 and tau inhibitors inhibit this activity. SIRT4 inhibits CAC. SIRT5 inhibits PDH while SIRT3 activates PDH. ROS activates the Keap1, Nrf2 complex. Nrf2 dissociates and enters the nucleus, where it increases transcription for antioxidants to inhibit ROS. SOD, CAT, mGSH, and empagliflozin also inhibit ROS.

**Fig. 3:**
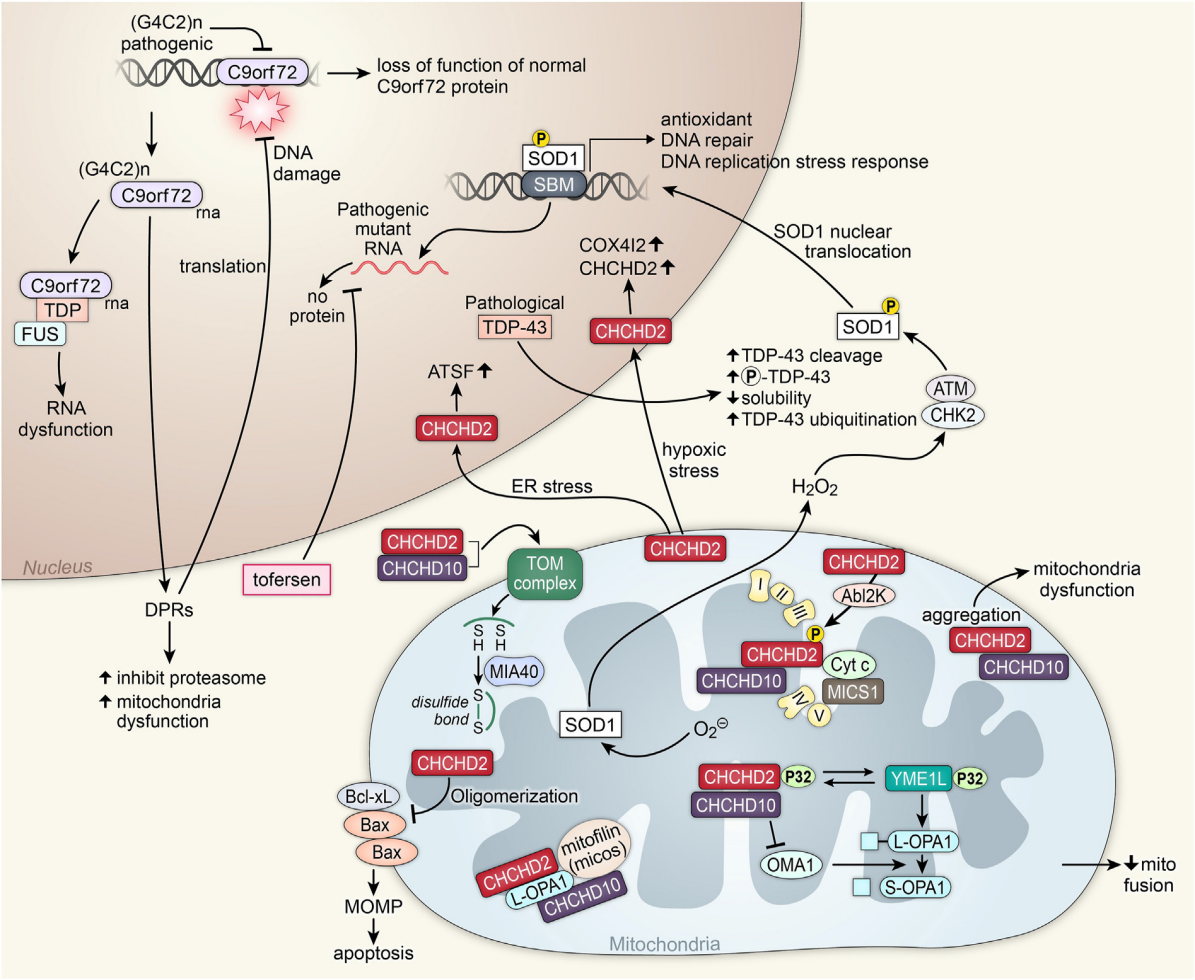
Amyotrophic lateral sclerosis. Above is a figure illustrating mitochondrial affecting pathways in ALS associated with CHCHD2, CHCHD10, C9orf72, TDP-43, and SOD1. Superoxide activates the SOD1 protein. SOD1, with hydrogen peroxide and a CHK2 ATM complex, is phosphorylated, which allows it to enter the nucleus through SOD1 nuclear translocation. In the nucleus, it functions as a transcription factor on SBM to transcript antioxidants proteins, DNA repair proteins, and proteins for the DNA replication stress response. Pathogenic SOD1 creating mutant RNA will produce no protein. Tofersen inhibits this process. Pathological TDP-43 leads to increased TDP-43 cleavage, increased phosphorylation of TDP-43, decreased solubility, and increased TDP-43 ubiquitination. Pathogenic (G4C2)n inhibits the expression of normal C9orf72, leading to the loss of function of normal C9orf72 protein. (G4C2)n C9orf72 RNA is transcribed, leading to DPRs that inhibit the proteasome and lead to mitochondrial dysfunction. DPRs also lead to DNA damage. The (G4C2)n C9orf72 RNA can also create a complex with TDP and FUS, leading to RNA dysfunction. With hypoxic stress, CHCHD2 will enter the nucleus and increase COX4I2 and CHCHD2. Under ER stress, CHCHD2 will enter the nucleus and increase ATSF. CHCHD2 works with AbI2k to phosphorylate a CHCHD2, CHCHD10, Cyt *c*, and MICS1 complex in the ETC. Oligomerization of CHCHD2 leads to inhibition of the Bcl-xL, Bax, and Bax complex, leading to MOMP and apoptosis. Aggregates of CHCHD2 and CHCHd10 in the mitochondria lead to mitochondrial dysfunction. A CHCHD2 and CHCHD10 complex activate a TOM complex, creating disulfide bonds with MIA40. A complex of CHCHD2, CHCHD10, and p32 transfers the p32 to YME1L. The YME1L p32 complex cleaves L-OPA1 to become S-OPA1 with OMA1. OMA1 is inhibited by the CHCHD2, CHCHD10, and p32 complex, decreasing mitochondrial fusion.

**Fig. 4:**
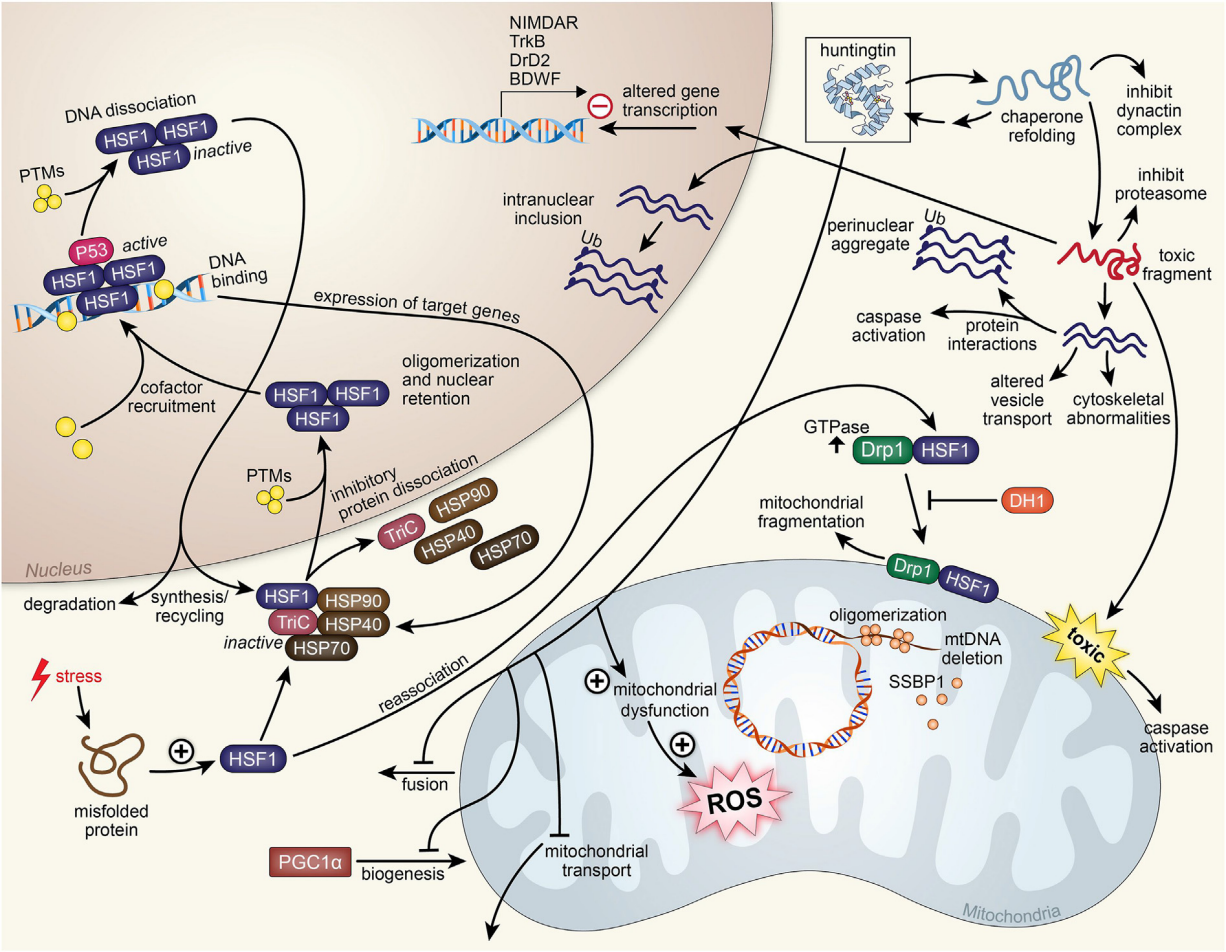
Huntington's disease. Above is a figure illustrating mitochondrial affecting pathways in Huntington's disease associated with huntingtin and HSF1. Huntingtin in the mitochondria leads to mitochondrial dysfunction, leading to the production of ROS. Huntingtin also inhibits mitochondrial transport, inhibits PGC1α mitochondrial biogenesis, and inhibits mitochondrial fusion. With chaperone refolding, huntingtin can inhibit the dynactin complex. This refold can also become a toxic fragment that can inhibit the proteasome, and is also toxic to the mitochondria, leading to caspase activation. The toxic fragment can enter the mitochondria and negatively alter gene transcription, specifically affecting genes NIMDAR, TrkB, DrD2, and BDWF. In the nucleus, the toxic fragment can further associate with more fragments and create an intranuclear inclusion. The association of multiple toxic fragments can lead to cytoskeletal abnormalities and altered vesicle transport. These fragments can interact with proteins and lead to caspase activation. The association of more toxic fragments creates a perinuclear aggregate. Stress in the cell causing a misfolded protein causes the increase of HSF1. HSF1 can reassociate with Drp1 and in high GTPase conditions associate with mitochondria and lead to fragmentation. HSF1 with DRP1 allows mtDNA deletion by SSBP1. DH1 inhibits this process. HSF1 can form an inactive complex with proteins TriC, HSP70, HSP40, and HSP90 that allows it to travel into the nucleus where it dissociates with that complex and oligomerizes with other HSF1 proteins and is modified with PTMs for nuclear retention. With cofactors and P53, the HSF1 oligomer actively binds to DNA and expresses target genes TriC, HSP70, HSP40, and HSP90. Inhibitory PTMs allow the HSF1 oligomer to dissociate from DNA where it is either degraded in the cytoplasm or recycled for further synthesis of target genes.

**Fig. 5:**
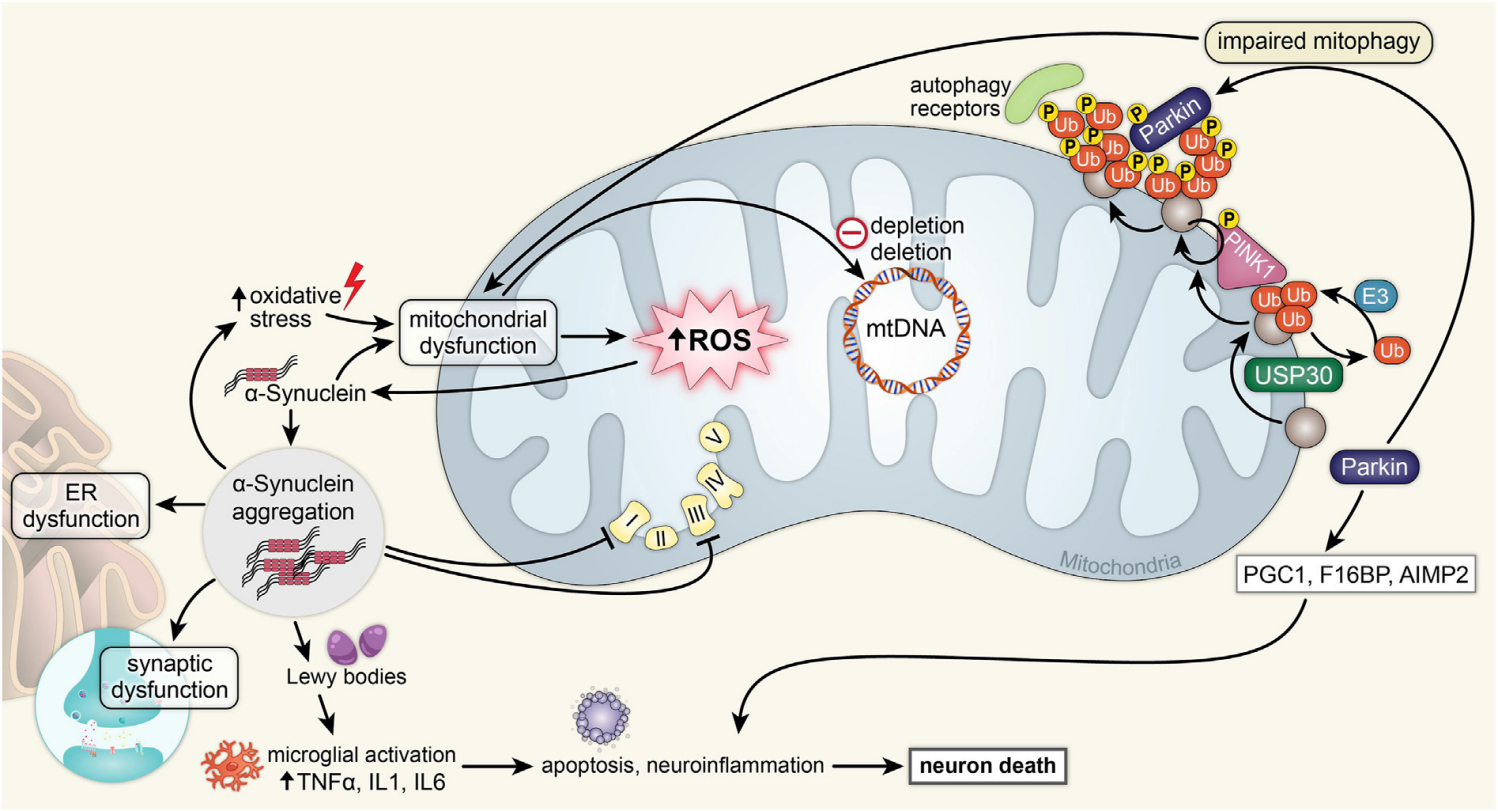
Parkinson's disease. Above is a figure illustrating mitochondrial affecting pathways in Parkinson's associated with α-synuclein and Parkin. Increased oxidative stress leads to mitochondrial dysfunction, which leads to increased mitochondrial ROS and mitochondrial DNA depletion and deletion. Mitochondrial ROS activate α-synuclein, leading to α-synuclein aggregation in the cytoplasm. These aggregates cause ER dysfunction, synaptic dysfunction, inhibition of ETC complexes 1 and 3, and the formation of Lewy bodies. Lewy bodies cause microglial activation by increasing TNFα, IL1, and IL6, which leads to apoptosis, neuroinflammation, and neuron death. Parkin leads to impaired mitophagy involving both Parkin and PINK1, leading to mitochondrial dysfunction. Parkin also leads to the increase of PGC1, F1GBP, and AIMP2, which leads to neuroinflammation and neuron death.

### Structure and function of mitochondria

Mitochondria are independent organelles, typically having an ellipsoid shape. Every aspect of the mitochondria's form is linked to a highly specialized function, with dynamic events allowing their appearance to range from their more typically described rod shape to more complex reticular networks [5]. A healthy cell can be judged by the shape of its mitochondria [4]. The porous outer membrane serves as a boundary between the inside of the mitochondria and the host cytoplasm. Its composition is the same as the cell lipid membrane, allowing the diffusion of lipid-soluble molecules into the intermembrane space. It contains many important enzymes and receptors [6]. Its porous nature makes it permeable to small molecules and ions. The voltage-dependent anion channel in the outer membrane allows transport of both hydrophilic and small proteins (less than 5000 ​Da). In many cases, mitochondria form a complex reticulum that interacts with other cellular components such as the cytoskeleton and endoplasmic reticulum (ER). Even though these interactions are poorly understood at the molecular level, they appear to underlie mitochondrial dynamics and replication, as well as their involvement in processes such as calcium homeostasis [5]. Surrounding the central matrix of the mitochondria is the inner membrane, which is impermeable to polar molecules and ions. The inner membrane is convoluted and invaginated [7]. This membrane is enriched with cardiolipin and contains more proteins than the outer membrane, including elevated protein levels required for a variety of biochemical pathways, including oxidative phosphorylation. The inner mitochondrial membrane is impermeable to most small ions and molecules, including H+, which is especially important in oxidative phosphorylation [7]. The intermembrane space is a result of the impermeability of the inner membrane and the highly permeable outer membrane. The environment of the intermembrane space is like the cytoplasm but with higher specificity for larger mitochondrial proteins. Due to its heavy impact on mitochondrial function, it serves as a limiting factor when targeting the mitochondria with drug therapies. There's evidence that the mitochondrial inner membrane is a dynamic structure that's able to change its shape rapidly in response to changes in osmotic and metabolic conditions [5].

Mitochondria contain their own genome, which is unique amongst organelles of animal cells. However, this genome only encodes 13 proteins of the respiratory chain [8]. A cell's nucleus carries just one set of DNA, while each mammalian mitochondrion contains 2–10 copies of mtDNA, resulting in 1000–100,000 copies in each human cell [9]. Individual mtDNA molecules replicate at random, and if there are two or more different types of mtDNA in a cell, any one type may replicate more frequently than the other [9]. If one set were to not function, another could make up for it by continuing to express that protein. However, the number of copies of mitochondrial DNA is not the same for everyone, with a smaller number of copies indicating reduced efficiency in the body and brain. Because of the limited number of proteins encoded in their DNA, mitochondria need the nucleus for the production and transport of the majority of their components. Larger proteins require specific mitochondrial targeting sequences to access them. Two transport proteins govern access to different compartments of the organelle: the translocator of the outer membrane (TOM) and the translocator of the inner membrane (TIM). The signal sequence is recognized by a receptor protein in the TOM complex and inserted with adjacent polypeptides. The polypeptide interacting with TIM can enter the matrix or move laterally inside the inner membrane itself [8]. The mitochondrial intermembrane space assembly (MIA) complex imports and folds many intermembrane space proteins.[10,11]. Oxidase assembly machinery (OXA) is required for some proteins that are synthesized on the matrix ribosomes to be exported to the inner membrane [8].

Electron tomography analysis confirmed that cristae arise from a distinct membrane, connecting through the intermembrane space through tubular junctions. Depending on the conformational state, cristae can vary from a simple tubular structure to a more complex lamellar structure that merges with the inner boundary membrane with 28-nm tubular structures. The cristae have a large surface area for oxidative phosphorylation and maintenance of the proton gradient, illustrating the highly adapted mitochondria. The complexity of cristae can vary by tissue, cell type, developmental stage, or physiological condition [5]. EM chromatography strongly suggests that diffusion between these internal compartments is highly restricted. Since oxidative phosphorylation relies on the rapid diffusion of ions and substrates on the inner mitochondrial membrane, the conformation of crista junctions could regulate rates of ATP phosphorylation. Similarly, the conformation of cristae can be expected to regulate rates of redox reactions involving cytochrome *c* by influencing its diffusion. If it's true that cristae morphology can regulate the rate of chemiosmosis, then they may be a key part of feedback mechanisms in response to environmental change [5]. ATP synthase is responsible for most cell energy production, cellular bioenergetics, and inner membrane structure [12]. Subdomains have been identified in the cristae with many specific mitochondrial proteins, notably OXPHOS proteins [13]. Due to its adaptive qualities, we likely don't completely understand the crucial role that cristae play in mitochondrial maintenance. Mitochondria's presence in every nucleated eukaryotic cell illustrates its necessity for a variety of functions. These mitochondrial functions are often oversimplified, with these organelles being referred to as the ‘powerhouses’ of the cell due to their well-known role in ATP production via oxidative phosphorylation [14]. In addition, mitochondria are responsible for calcium handling, apoptosis, cell signaling, and ROS production.

### Discovery of mitochondrial dysfunction in neurodegeneration: seminal discoveries in the past three decades linking mitochondrial dysfunction to the etiopathogenesis of neurodegenerative disorders

Mitochondrial disease was discovered by Luft and colleagues [15] in 1962 in a non-thyroidal hypermetabolism case. Major advances in both the understanding of mitochondria and biology have been made since, and a number of mitochondrial disorders have been recognized (Fig. 1) [16]. Mitochondrial diseases arise from the dysfunction of the mitochondrial respiratory chain that occurs due to mutations in either the mitochondrial or nuclear genome. They can be classified as primary disorders, meaning they arise from mtDNA defects, or secondary disorders, meaning they are caused by a failure in intergenomic signaling leading to an accumulation in mtDNA deletion. These mtDNA defects and respiratory chain abnormalities are linked to the pathogenesis of many neurodegenerative disorders, including Parkinson's disease [17]. Mitochondrial disease is consistently linked with neurological deficits and is often disabling [18]. There are many reasons why the central nervous system (CNS) is particularly vulnerable to mitochondrial dysfunction. First, the brain is highly metabolically active and therefore susceptible to bioenergetic failure [19]. Second, the brain has fewer antioxidant defenses against ROS than other tissues [20]. Third, most neurons within the brain are post-mitotic and irreplaceable (except for the subventricular zone, olfactory epithelium, and hippocampus) [21]. Any neuronal injury will prove fatal to the cell if it is not alleviated in some way. Neurons have a consistently high demand for ATP produced via mitochondrial metabolism. This is reflected by the high mitochondrial mass in the neuronal cell body, axon, presynaptic terminals, and dendritic branches [22]. To support this metabolic demand, it's necessary for mitochondria to be highly dynamic, where they continuously move, fuse, and divide. Mitochondria are essential for ATP generation, but they also play essential roles in the production of iron-sulfur clusters, calcium handling, cell death, and ROS signaling. All these processes, to some extent, have been connected to neurogenerative diseases.

The mitochondrial theory of aging arose in 1972. This stated that somatic mtDNA mutations generated excessive ROS, and these further damaged mtDNA, creating a continuous feedforward loop of damage. It is suggested that the maximal lifespan of a given mammalian species is largely an expression of genetic control over the rate of oxygen utilization, which determines the rate of damage accumulation produced by free radicals in the mitochondria. This damage increases with an increased rate of oxygen consumption [23]. Historically, the first connection thought to exist between neurogenerative disorders and mitochondrial diseases was in Parkinson's disease (PD). It was discovered in the 1980s that exposure to the neurotoxin 1-methyl-4-phenyl-1,2,3,6-tetrahydropyridine (MPTP) via illicit drug use led to acute parkinsonian syndrome that was clinically indistinguishable from PD [24]. This PD-like condition was a result of an electron flow blockage in complex 1 in the mitochondrial ETC [25,26]. Reports that MPTP and other complex 1 inhibitors produce PD features in rodents strengthened the idea that complex 1 inhibition can cause PD-like neurodegeneration [[27], [28], [29]]. These discoveries led investigators to assess mitochondrial respiration in biospecimens from PD patients, where they found significant reductions in activity in the ETC, particularly in complex 1 of the brain [[30], [31], [32]]. This led to the conclusion that deficient Complex 1 function caused PD pathogenesis. The chronic use of levodopa, a widely used anti-PD therapy, was found to alter OxPhos activity in rodent brains [33]. Concurrently, a connection between the mitochondria and Alzheimer's emerged with reports of mitochondrial morphological alterations in postmortem brain sections 34,35], as well as metabolic alterations in fibroblasts from patients, including reduced glucose and deficits in calcium homeostasis [36]. It was hypothesized that, with these damages and neuronal vulnerability, mitochondrial dysfunction was the cause of the behavioral deficits in AD. Cytoplasmic hybrids repopulated with mitochondria from AD patients displayed mitochondrial alterations [37]. There were also reductions in Complex 5 activity [38], as well as Complex 1 and Complex 4 deficiencies in platelets and brain tissue [39,40]. However, it was pointed out that although complex 4 deficiency could be behind AD pathogenesis, similar reductions in complex 4 were observed in other neurodegenerative diseases [41]. This implies that a deficiency in complex 4 is likely a non-AD-specific change. Also, exposing isolated mitochondria to amyloid-B oligomers, key actors in AD, resulted in mitochondrial dysfunction [42]. Subsequently, it was discovered that there is a mitochondrial metabolic enzyme deficiency in Alzheimer's disease. PDHC activity was found to be significantly reduced in Alzheimer's disease in the frontal cortex (98). Thereafter, it was identified that there were deficiencies in the respiratory chain in individuals with Alzheimer's disease. The mean cytochrome oxidase activity in Alzheimer's patients was found to be reduced in the frontal, temporal, and parietal cortices compared to healthy controls.

A couple of decades ago, it was discovered that HTT binds to huntingtin interacting protein 1 (HAP1), which is a cytosolic protein that associates with microtubules and other membranous compartments of the cell, including mitochondria. HAP1 interacts with the p150-glued subunit of dynactin (DYNC1LI2) and PCM-1. Both HAP1 and p150Glued are highly expressed in neurons. These interactions may explain why microtubules are destabilized in Huntington's disease [43]. It was also reported that there were respiratory chain abnormalities in the spinal cords of sporadic ALS patients. There was found to be a severe loss of myelinated axons associated with irregularities in the myelin sheath. Abnormalities in the myelin sheath are comparable to those reported in patients with mutations located in the extracellular domain of myelin P0, as well as in myelin protein P0-deficient mice [44]. Following this, in 2004, it was determined that 20 ​% of those with familial ALS have mutations in the Cu–Zn superoxide dismutase 1 (SOD1) gene, which is present in both the cytosol and the intermembrane space. There was a preferential association of the mutant SOD1 with spinal cord mitochondria [45]. With this mutation, there was increased oxidative damage [46]. In the same year, a correlation was discovered between the formation of mtROS, nitrogen species (RONS), rates of neurodegeneration in the brain and retina, and maximum lifespan potentials in five different mammalian species. Mitochondria are the primary source of RONS formation, and these species are involved in the intrinsic apoptosis pathway [47]. Also in 2004, it was discovered that Huntington's disease is caused by the abnormal expansion of a CAG repeat in the Huntington's disease gene on chromosome 4, which encodes huntingtin (HTT). The Huntingtin protein is located on the cytosolic surface of the outer mitochondrial membrane. The mutant huntingtin N-terminus increases susceptibility to calcium-induced MPT opening, leading to mitochondrial swelling and MPT-dependent cytochrome *c* release. There was a decrease in complex 2 and complex 3 function in postmortem Huntington's disease brains. Inhibition of complex 2 by malonate in experimental animals caused Huntington's disease-like pathogenic lesions [48]. In 2006, it was confirmed by the age-related accumulation of somatic and clonal mtDNA deletions in the substantia nigra that neurons with high mutation loads were COX-negative. These mutations cause respiratory chain deficiency. This was true for both older controls and individuals with Parkinson's disease. This suggests that somatic mtDNA deletions are important in the selective neuronal loss observed in both brain aging and Parkinson's disease [49]. In the same year, it was also determined that different mtDNA haplotypes may modulate oxidative phosphorylation, possibly predisposing them to or protecting them from certain diseases [50]. Also this year, the following proteins were found to have implications for sporadic Parkinson's disease: PARK2 (encodes parkin), PINK1 (encodes PTEN-inducing putative kinase 1), PARK7 (encodes DJ-1), SNCA (encodes alpha-synuclein), LRRK2 (encodes dardarin), and HTRA2 (encodes Omi/HTRA2) [51]. Also, in relation to Parkinson's, it was determined that autosomal recessive parkinsonism is not uncommon in patients with PEO and mutations in POLG and can be seen even in young patients without PEO [52]. In addition, it was also found that nonglycosylated full-length C-terminal truncated APP proteins accumulate exclusively in the mitochondrial protein import channel in Alzheimer's brains but not in age-related controls. Interactions between various mitochondrial translocating proteins are expected to be transient. In contrast, the interactions between APP and mitochondrial translocases are stable and persistent in the Alzheimer's brain. The accumulation of APP in mitochondrial protein channels may inhibit the import of proteins essential for normal mitochondrial function. Consistent with this possibility, it was found that accumulation of APP in import channels inhibited the import of cytochrome *c* oxidase subunits 4 and 5b, which caused a decrease in cytochrome *c* oxidase activity and mitochondrial dysfunction [53]. In 2007, it was reported that mutant HTT impairs mitochondrial function by repressing PGC-1-regulated gene transcription of many nuclei-encoded mitochondrial genes, including subunits of the ETC. Expression is repressed through promoter binding and interference with CREB-dependent transcription. Cells that do not express PGC-1 have an impaired ROS defense system because PGC-1-regulated antioxidant systems are reduced. Overexpression of PGC-1 improves atrophy of striatal neurons in transgenic mice with Huntington's [54]. Also in 2007, it was found that mutations in CoQ10 are likely associated with encephalomyopathic syndromes. Of the 9 genes involved in CoQ10 biosynthesis and suspected of causing primary CoQ10 deficiency, 3 have been found to do so: PDSS1, PDSS2, and COQ2. Some of these patients may be helped by the timely administration of high-dose CoQ10 [55]. The sequencing of mtDNA has uncovered innumerable pathogenic mutations, reaching 281 in 2018 [56,57].

## Mechanistic insights to mitochondrial dysfunction in neurodegeneration

### Electron transport chain and oxidative phosphorylation

The energy demands of the cell are reflected in the number of mitochondria it contains. Energy-demanding tissues such as muscle, cardiomyocytes, and neurons tend to have more mitochondria. Energy is harnessed by first transporting electrons between a chain of inner mitochondrial membrane proteins encoded in both mitochondrial and nuclear genomes. Recent data suggests complexes 1, 3, and 4, can be organized into supercomplexes, supporting more efficient substrate movement [58,59].

Complex 1 is the first and largest complex of the electron transport chain, composed of over 40 subunits with a combined molecular weight of 908 ​kDa [60,61]. Seven subunits are encoded in the mitochondrial genome (MT-ND1-6 and MT-ND4L) and the rest are encoded in the nucleus [60,61]. Complex 1 catalyzes the oxidation of NADH from the citric acid cycle, yielding two electrons that first pass to a flavin mononucleotide, and then through a series of iron-sulfur clusters to reduce ubiquinone to ubiquinol. Electron transfer in the ETC is coupled with the translocation of four protons across the inner mitochondrial membrane. Complex 2 is a succinate/ubiquinone oxidoreductase, which oxidizes succinate to fumarate in the citric acid cycle. It consists of only 4 proteins all encoded from the nuclear genome [2]. Resulting electrons reduce ubiquinone in the ETC. Complex 2 is the smallest complex in the ETC and is encoded entirely by the nuclear genome. Protons are not translocated through this complex [62].

The reoxidation of ubiquinone is catalyzed by ubiquinol/cytochrome *c* oxidoreductase in complex 3, which is composed of 11 subunits entirely encoded by the nucleus except for cytochrome *b*. Ubiquinol oxidation releases the two electrons given from complexes 1 and 2. The electron transfer from ubiquinol to cytochrome *c* consists of 2 steps. First, one electron is transferred to the iron-sulfur cluster, then to cytochrome *c* via cytochrome cl. The second electron is recycled to ubiquinol in a reaction called the Q-cycle. In this reaction, two ubiquinols are oxidized by two electron transfers, reducing one ubiquinone. In this whole process, each electron transferred results in 2 proton translocations [1].

Finally, electrons reach complex 4 (cytochrome *c* oxidase), which contains 13 subunits, 3 encoded by the mitochondrial genome (COX1, 2, 3), and the other 10 encoded by nuclear [2]. The substrate for this complex is cytochrome *c*, transferring electrons between complexes 3 and 4. Cytochrome *c* is a hemoprotein, and the key enzyme in the overall regulation of the ETC [2]. It works by transferring electrons one by one in different ‘states’ to complex 4. Each electron transfer results in one proton translocation across the membrane, totaling 8 proton translocations as electrons as passed to oxygen, generating a proton gradient. This proton gradient is utilized by the final complex of oxidative complex machinery to generate ATP [63].

Complex 5, or ATP synthase, is a large multi-subunit complex containing two subunits encoded by the mitochondrial genome (ATPases 6 and 8), and 11 subunits encoded by nuclear DNA [2]. It has 2 main domains: F0, which is embedded in the inner mitochondrial membrane, and F1, the catalytic domain, which lies on the matrix side of the inner membrane in regions of high membrane curvature [14,64,65]. ATP synthase is a dimer, and there is a strong dependence on cristae structure and ATP synthase supercomplex formation [14]. Because of the proton gradient, protons can diffuse across the inner membrane from the inner membrane space, promoting the rotation of subunit F1. The B subunits of F1 transform between 3 conformational states, and one proton is required for each subunit state. Energy harnessed during the rotation of F1 is used to synthesize ATP, which is released in the following rotation. Each ATP requires the movement of 3 protons [64,65].

ATP generation through oxidative phosphorylation is an extremely efficient method of aerobic respiration, which is the heart of energy metabolism for animals, plants, and most microbial life forms. It is much more efficient than substrate-level phosphorylation from glycolysis alone. Therefore, there's no doubt a defect that affects the ability of the mitochondria to carry out this essential process will substantially affect the production of ATP and be detrimental to cell functioning. The generation of ATP (phosphorylation) is tightly coupled with the proton motive force across the inner mitochondrial membrane [14].

### Mitochondria in cell death

Apoptosis is a form of cell death associated with the mitochondria that is highly regulated. It is one of four forms of cell death (apoptotic, necrotic, autophagic, parthanatos) that are all important in mitochondrial associated neurodegenerative disease. The least understood of these pathways is autophagic. Although the exact mechanism of this pathway is still to be determined, it is known that cells lost to this pathwasy show an accumulation of autophagosomes. Recent work has suggested that autophagy may be mediated through sodium/potassium ATPase[ 66].

Apoptosis is often the result of intracellular signaling that leads to cell shrinkage, membrane blebbing, nuclear fragmentation, and condensation of nuclear chromatin. Following these morphological changes, the cell will be systematically dismantled and degraded. It was originally assumed this was controlled at the nuclear level, however, apoptosis occurs normally in enucleated cells, meaning apoptosis must be regulated at the cytoplasmic level [67]. The 2 main apoptotic pathways that occur in mammalian cells are the intrinsic and extrinsic pathways, in which the mitochondria play an essential role. The intrinsic pathway is controlled by the Bcl-2 family, directing death signaling to the mitochondria which facilitates the release of pro-apoptotic proteins from the intermembrane space [68]. The extrinsic pathway activates caspase-8 along with the formation of the death inducible signaling complex, which initiates a cascade of protein interactions leading to the permeabilization of the mitochondrial outer membrane and cytochrome *c* release from the intermembrane space [68,69]. The main effectors of apoptosis are antiapoptotic Bcl-2 proteins, proapoptotic Bcl-2 inhibitor BH3 proteins, and proapoptotic BAX and BAK, which interact with the mitochondria. Bcl-2 proteins are positive and negative regulators of BAX/BAK respectively [67]. The loss of these proteins in cells leads to resistance to apoptotic stimuli.

Mitochondria play a crucial role in apoptosis by releasing cytochrome *c* from the intermembrane space, which activates the caspases that initiate apoptosis. Two mechanisms have been proposed for this release. First, the opening of a high-conductance channel in the inner membrane collapses the membrane potential, which leads to a mitochondrial permeability transition (MPT) [4]. This leads to swelling of the matrix and the rupture of the outer membrane. The second mechanism involves the transport of cytochrome *c* across a specific yet unidentified pore in the outer membrane [4]. Electron microscopy and tomography of mitochondria in a cell-free model from Xenopus eggs have suggested that cytochrome *c* can be released upon characteristic apoptotic activation (caspase activation) without swelling of the matrix or ruptures in the outer membrane [4]. These mitochondria continued to import proteins and did not experience a permeability transition. It's possible that MPT is not a cause but rather a consequence of apoptosis in the mitochondria [4].

Necrosis occurs in response to extracellular stimuli, ischemia, or trauma, leading to cellular swelling, depletion of energy stores, and the disruption of cellular membranes. In response to ischemic injury, such as one that lowers pH in response to anaerobic conditions and a depletion of ATP, calcium intake is prevented by the mitochondria, increasing intracellular calcium. The changes in calcium levels trigger the opening of the mitochondrial permeability transition pore (mPTP). The mPTP is a channel complex composed of different proteins. The exact structure is unknown, but some proteins identified include VDAC, ANT, and cyclophilin D [[69], [70], [71], [72]]. When the pore opens, the permeability of the inner mitochondrial membrane dramatically changes, which may be irreversible if prolonged. These changes in permeability deplete the mitochondrial membrane potential, disrupt oxidative phosphorylation, and cause swelling of the matrix. The outer membrane ruptures, activating a hydrolytic enzymes, leading to necrosis. However, due to the rupture of the mitochondria, the release of pro-apoptotic proteins will also occur, so it's likely that the opening of mPTP will not solely lead to necrosis [[69], [70], [71], [72]]. Cells lacking most of their mitochondria are still susceptible to programmed necrosis, indicating that mitochondria or their metabolism may not be necessary for necrosis execution [73].

Parthanatos is mediated by poly ADP ribose polymerase-1 (PARP-1). DNA damage, ischemic injury, and DNA-damaging agents including ROS and ionizing radiation activates PARP-1 [[74], [75], [76]]. Parthanatos differs from other forms of cell death because it occurs through the externalization of phosphatidylserine onto the outer cell membrane, causing a loss of mitochondrial membrane potential and DNA fragmentation. No cellular swelling occurs, but the membrane integrity is lost, as well as the release of mitochondrial apoptosis-inducing factors. In addition, activated PARP-1 forms poly ADP ribose polymers from nicotinamide adenine dinucleotide (NAD), meaning the overactivation of PARP-1 can deplete NAD and ATP [[74], [75], [76]]. Pharmacologically, inhibiting or genetically knocking down PARP-1 provides cytoprotection, indicating that PARP-1 plays a significant role following cellular injury [77].

Mitochondrial dysfunction is likely one of the number of factors involved in the cell death that's a part of neurodegeneration. What is clear is that changes in this organelle's function will have a great impact on the survival and functioning of these cells.

### Mitochondria and calcium handling

Mitochondria play a key role in the buffering and maintenance of cellular calcium levels. These organelles have a large capacity for calcium through the expression of a uniporter in the inner mitochondrial membrane. This uniporter allows calcium to enter the mitochondria down its electrochemical gradient. Its modular proteins include MICU1, MICU2, MICU3, and EMRE [[78], [79], [80], [81]]. Sodium and calcium ion exchange mediate the efflux of calcium from the mitochondria [82,83], ensuring that equilibrium is never reached. In theory, mitochondria are consistently in an unlimited calcium drain. Calcium transport across the outer mitochondrial membrane occurs through VDAC, the most abundant protein in the outer membrane. The expression of this protein directly correlates with the rapid influx of calcium into the mitochondria [[84], [85], [86]]. Calcium ions also serve as secondary messengers in a variety of signaling pathways, are important in neurotransmitter release, and help regulate gene expression [87]. It's possible that the ability of the mitochondria to take up calcium and act as a spatial buffer in the cell [[88], [89], [90]] may have an impact on spatiotemporal calcium signaling characteristics, shaping the activation of downstream targets. Calcium transport from the cytosol to the mitochondrial matrix is also important in energy homeostasis. Increasing Ca^2+^ influx activates pyruvate isocitrate and oxoglutarate dehydrogenase, which activate rate-limiting enzymes of the Krebs cycle. This ultimately increases ATP production [91,92]. Both calcium signaling and mitochondrial calcium intake are linked to apoptosis and necrosis through the mitochondrial permeability transition pore (mPTP). An overload of Ca^2+^ induces the opening of the pore, which leads to calcium-dependent necrotic cell death. The opening of the pore causes the collapse of the mitochondrial membrane potential, leading to bioenergetic failure.

### Reactive oxygen species and mitochondrial homeostasis

In the process of energy production, several waste products are produced such as carbon dioxide that we exhale, water that we excrete in the urine, and free radicals, or reactive oxygen species (ROS), which are highly corrosive [4]. In normal conditions, 1–5% of oxygen is converted to ROS primarily from complexes 1 and 3 of the ETC [93,94]. However, excessive ROS production damages a variety of cellular components including proteins, lipids, and DNA. One-fifth of inspired oxygen is used by the brain, 90 ​% of this being consumed by oxidative phosphorylation. Since neurons have high oxidative metabolic activity, a non-replicative nature, and a relatively low antioxidant capacity, they are highly susceptible to the damaging effects of ROS. ROS production is dependent on the release of electrons out of the ETC [3], which produce superoxides. These can be converted to other reactive species, including hydrogen peroxide and peroxynitrite, through superoxide dismutase 2 (SOD2) or NO respectively. ROS may be damaging, but they also serve as important signaling molecules for protein expression and signaling cascades. For example, ROS signaling is important for O_2_ sensing during hypoxia. Superoxide production increases in hypoxic conditions and the superoxide is converted to hydrogen peroxide in the matrix before diffusion to the cytoplasm. The hydrogen peroxide stabilizes hypoxia-inducible factor 1α (HIF1α) to properly transcribe machinery to respond to hypoxia. Mitochondrial calcium uptake can also be regulated by ROS. A prolonged elevation of ROS in the mitochondria is involved in several processes including cell proliferation. However, overproduction of ROS or a dysregulation of the antioxidant system can lead to several pathologies, including cell death and neurodegeneration [3]. It is in a feedforward loop where mitochondrial dysfunction leads to an increase in the production of ROS, damaging cellular components, while also causing further mitochondrial dysfunction. ROS production induces damage to the ETC through complexes 1 and 3, which increases the electron reduction of oxygen to additional ROS. This is a vicious cycle of ROS production and organelle dysregulation that ultimately leads to apoptosis [95]. Even acute ROS exposure can also inactivate iron-sulfur clusters, ultimately shutting down mitochondrial energy production [96]. ROS may also promote a mitochondrial permeability transition by oxidizing thiol groups in part of the mitochondrial permeability transition pore [97].

Excessive ROS generation can alter calcium homeostasis by damaging calcium transport proteins and triggering calcium release from the mitochondria [98,99]. Elevation of calcium causes a change in mitochondrial potential and leads to the production of additional superoxide ion radicals [100]. If the mitochondrial becomes overloaded with calcium, it undergoes a permeability transition, resulting in a rupture of the outer mitochondrial membrane [100]. Sustained elevations in intracellular calcium can cause neurodegeneration [6]. Normally, mitochondria are protected from oxidative damage by a network of antioxidants consisting of superoxide dismutase, catalase, glutathione peroxidase, glutathione reductase, α-tocopherol, and ubiquinol [93,101]. However, this system is not perfect, and the system cannot fully neutralize ROS being emitted from the mitochondria. Cumulative oxidative injuries to the mitochondria cause progressive loss of efficiency, which causes a greater proportion of oxygen to be converted to ROS, which continues to undermine the defense system [102].

In the context of aging, the ROS theory suggests that over time, the accumulation of oxidative damage overwhelms the cellular defense mechanisms, leading to the progressive decline and dysfunction of tissues and organs. This oxidative damage can result in impaired cellular function, increased inflammation, and DNA mutations, which can further contribute to the aging process. In the case of neuropathology, oxidative stress plays a significant role in the development and progression of neurodegenerative disease. This ties together the notion that much of the cause of aging relates back to inflammatory processes [[103], [104], [105]]. Furthermore, once in oversupply, ROS activates classical inflammatory responses and promotes the production of pro-inflammatory signals, which triggers neuroinflammation [106,107]. Neuroinflammation is central to much of the damage associated with disease pathology and is additive to the foldopathy contributors to the disease state [108,109]. What are the causative factors of an increase in ROS in the body over time? One theory points to the accumulation of total amounts of iron in the body [109,110]. The human body has limited ways to shed iron and does an admirable job of holding onto stores of reactive metal. This makes evolutionary sense, since the absorption of iron from the diet is rate-limited, and the process of erythropoiesis to replace lost blood due to injury is an iron-critical process. The ability to regenerate blood supply was vital in a more primitive world. As civilization has reduced the number of times a blood loss event occurs to most individuals, coupled with a diet heavy in iron-rich/iron-fortified foods, humans show a marked increase in total body iron in later life [111,112]. The level of iron leads to an increase in serum ferritin. It has been noted that neurodegeneration diseases are accompanied by elevated ferritin counts, but the amount of serum ferritin increasing with age is considered “normal”. This itself is curious, as ferritin is primarily an intracellular protein, and the release into the circulatory system is itself an indicator of cellular disruption. Macrophages are tasked with the collection of poorly chelated iron, and the presence of iron-laden macrophages is another hallmark noted in individuals prone to neurodegenerative disease [[113], [114], [115]].

#### Effect of ROS on telomeres

ROS has been reported to shorten telomere caps, which are specialized structures found at the ends of chromosomes. Telomeres play a crucial role in maintaining genomic stability and protecting the integrity of the DNA during cell division. They act as protective caps that prevent the ends of chromosomes from being recognized as damaged DNA and triggering a cellular response. Telomeres consist of repetitive DNA sequences and associated proteins, forming a complex structure that prevents the degradation and fusion of chromosome ends. However, telomeres naturally undergo shortening with each round of cell division, as the DNA replication machinery cannot fully replicate the very ends of chromosomes. This gradual telomere shortening is a normal part of the aging process [[116], [117], [118]]. ROS causes telomere shortening by oxidative damage to the DNA within telomeres. The repetitive DNA sequences that compose telomeres are particularly susceptible to oxidative stress due to their high guanine content. Guanine is vulnerable to oxidation, leading to the formation of DNA lesions, such as 8-oxoguanine [119,120]. When telomeric DNA is damaged by ROS-induced oxidative stress, it can impair the replication and maintenance of telomeres. This oxidative damage can accelerate telomere shortening and compromise the protective function of telomeres. As telomeres become critically short, they may trigger cellular senescence or apoptosis, leading to impaired tissue function and contributing to the aging process. This senescence is particularly impactful in post-mitotic cells.

#### Effect of ROS on the epigenome

Oxidative stress induced by ROS directly influences epigenetic modifications. As ROS oxidizes and damages DNA, it results in the formation of DNA lesions. These DNA lesions can disrupt the normal process of DNA methylation, which is a key epigenetic modification involved in gene regulation. DNA methylation patterns are altered in response to oxidative stress, which leads to changes in gene expression and age-related diseases. Further, oxidative stress (ROS) inhibits the activity of DNA methyltransferases (DNMTs), the enzymes responsible for adding methyl groups to DNA. This inhibition of DNMTs can lead to global DNA hypomethylation or alterations in specific gene regions, thereby influencing gene expression patterns [121,122].

DNMTs are a family of enzymes responsible for adding methyl groups to DNA, resulting in DNA methylation. DNMT1 is involved in maintenance methylation, while DNMT3A and DNMT3B are responsible for de novo DNA methylation. Genetic factors, such as transcription factors and signaling molecules, can activate or repress the expression or activity of DNMTs, thereby influencing DNA methylation patterns. ROS also impacts Ten-eleven Translocation (TET) enzymes that are involved in the process of DNA demethylation. They catalyze the conversion of 5-methylcytosine (5 ​mC) to 5-hydroxymethylcytosine (5hmC) and further oxidized derivatives [123]. TET enzymes are regulated by various genetic factors, including transcription factors and signaling pathways, which can modulate their expression and activity. ROS and oxidative stress also impact histone modifications, another crucial component of epigenetic regulation. Histone proteins can undergo oxidative modifications, such as the oxidation of specific amino acids within their structure. These oxidative modifications alter the structure and function of chromatin, leading to changes in gene expression patterns. Additionally, oxidative stress impacts the activity of the enzymes involved in histone modifications, further influencing the epigenetic landscape.

Histone modifying enzymes, including histone acetyltransferases (HATs), histone deacetylases (HDACs), histone methyltransferases (HMTs), and histone demethylases (HDMs), regulate the addition or removal of various chemical groups (e.g., acetyl, methyl) on histone proteins, influencing chromatin structure and gene expression [124]. The activity of these enzymes can be modulated by genetic factors, such as transcription factors and signaling pathways, that control their expression, localization, or enzymatic activity.

### Mitochondrial endoplasmic reticulum contacts

In 1980, it was determined that a maximum of 80 ​% of the mitochondria is in contact with the rough ER [82]. Mitochondrial-ER contacts (MERCs) are one of the most widespread organelle contacts. These sites range from 14 to 20 ​nm for the RER and 9–16 ​nm for the SER. Both the ER and mitochondria play key roles in transmitting Ca^2+^ signals in physiological and pathological conditions. This mitochondrial-associated membrane (MAM) between these organelles specializes in Ca^2+^ transfer. Calcium ion efflux from the ER crosses the outer mitochondrial membrane through VDAC channels, then reaches the inner mitochondrial membrane, accumulating in the matrix via the mitochondrial calcium uniporter (MCU). However, excessive accumulation of Ca^2+^ can activate the release of pro-apoptotic factors due to the opening of the mitochondrial permeability transition pore (mPTP). MAM is also a heavily involved domain in ER stress-mediated apoptosis. Both the mitochondria and ER are sites of ROS production, and MAM is a location where ROS exchange can occur. Many regulators of oxidative state are located at MERCs. MAM structure requires unique protein and lipid constituents to support formation, which is mostly made of cholesterol and sphingolipids to increase thickness, and a diverse set of enzymes for lipids trafficking and synthesis. MAM also contains enzymes for cholesterol and ceramide synthesis. Lipid composition is what is responsible for proper MAM activity. Defects in lipids metabolism, resulting in incorrect assembly and functioning of MAM sites, can lead to the onset and progression of various human neurodegenerative disorders [82].

### Reactive iron accumulation and lipid peroxidation: implications for the aging brain

Iron is an essential nutrient involved in various cellular processes, including energy production and neurotransmitter synthesis. However, excessive accumulation of iron and the generation of reactive iron species can lead to oxidative stress and brain damage. In the aging brain, there is evidence of increased iron accumulation, particularly in regions susceptible to neurodegenerative diseases, such as the substantia nigra in Parkinson's disease and the hippocampus in Alzheimer's disease. This iron accumulation can result from dysregulation of iron metabolism, impaired iron transport mechanisms, or age-related changes in iron-binding proteins. Reactive iron species can promote the generation of reactive oxygen species (ROS) through the Fenton reaction, where iron interacts with hydrogen peroxide. These ROS can cause oxidative damage to lipids, proteins, and DNA, leading to cellular dysfunction and neurodegeneration. Additionally, iron accumulation can disrupt cellular homeostasis by affecting mitochondrial function, impairing neurotransmitter release, promoting neuroinflammation, and influencing synaptic plasticity. These alterations contribute to the aging process and increase the susceptibility to neurodegenerative disorders.

Lipid peroxidation is a process involving the oxidative degradation of polyunsaturated fatty acids present in cell membranes. ROS, including those generated through iron-mediated reactions, can initiate lipid peroxidation. This process leads to the production of lipid peroxides and reactive aldehydes, such as malondialdehyde (MDA) and 4-hydroxynonenal (4-HNE). In the aging brain, lipid peroxidation levels tend to increase, particularly in vulnerable brain regions. This oxidative damage to lipids can disrupt the integrity and fluidity of cell membranes, impair neurotransmission, and compromise cellular functions. Lipid peroxidation products, such as 4-HNE, can also covalently modify proteins, altering their structure and function [125]. Furthermore, lipid peroxidation can activate inflammatory responses and promote the production of pro-inflammatory cytokines, further exacerbating neuroinflammation. Neuroinflammation is a common feature of aging and age-related neurodegenerative diseases and contributes to the progression of neuronal damage [126].

The combined effects of reactive iron accumulation and lipid peroxidation can create a vicious cycle in the aging brain. Reactive iron species can initiate lipid peroxidation, while lipid peroxidation products can, in turn, promote iron release from iron-binding proteins, fueling further oxidative stress and damage. Microglia move into the areas where this is occurring and take up the reactive iron. Once they are loaded with hemosiderin, they become classically activated, initiate toxic cytokine release, and further disrupt tissue integrity. This cycle of oxidative stress, lipid peroxidation, and iron accumulation has profound consequences for the aging brain, leading to neurodegeneration, cognitive decline, and increased susceptibility to neurodegenerative diseases. Strategies aimed at reducing iron accumulation, inhibiting lipid peroxidation.

### Lipofuscin accumulation is a marker of aging brain

Another telltale sign of iron accumulation in the body is an accumulation of lipofuscin. Lipofuscin, also known as age pigment, is a yellow-brown, autofluorescent substance that accumulates in cells over time, particularly in post-mitotic cells such as neurons and cardiac myocytes. It is composed of heavy metals, oxidized proteins, lipids, and other cellular components that have undergone oxidative damage and are not effectively cleared by cellular degradation mechanisms [127].

The accumulation of lipofuscin is considered a hallmark of aging and is often observed in various tissues, including the brain. It is believed to be a consequence of impaired lysosomal function and the diminished capacity of cells to degrade and remove damaged or dysfunctional components. One reported way to rapidly induce lipofuscin deposition in laboratory animals is to inject iron solution into the circulatory system. While this reliably forms a material that behaves like age-related lipofuscin in many respects, there is controversy with calling it “true” lipofuscin since it can be cleared by the body over time and lipofuscin is, by definition, insoluble and non-degradable [128].

In the context of the theory of aging, lipofuscin accumulation intersects with several factors. First, Lipofuscin contains oxidized molecules that result from the action of reactive oxygen species (ROS) on cellular components. ROS generated during normal cellular metabolism or due to various stressors can contribute to the accumulation of lipofuscin. Lipofuscin granules themselves can also generate ROS, creating a self-perpetuating cycle of oxidative damage [129]. Lipofuscin accumulation is considered a marker of oxidative stress and a consequence of cumulative oxidative damage over time. Oxidative stress leads to the generation of ROS and the oxidation of cellular components, including proteins and lipids, which can contribute to the formation of lipofuscin [130]. Lipofuscin accumulates within lysosomes, the cellular organelles responsible for degrading and recycling cellular waste and damaged materials. The impaired function of lysosomes, which can occur during aging, hinders the efficient breakdown of lipofuscin. As a result, lipofuscin gradually accumulates within lysosomes, leading to their dysfunction and further disruption of cellular homeostasis [130].

The accumulation of lipofuscin can interfere with cellular processes and contribute to cellular dysfunction. Lipofuscin-filled lysosomes can occupy space within the cell, potentially impairing organelle function and disrupting intracellular transport. Moreover, the presence of lipofuscin can interfere with the proper functioning of lysosomal enzymes and impair autophagy, the process by which cells remove damaged proteins and organelles [130,131]. While lipofuscin accumulation is associated with aging, its precise role in cellular and organismal senescence is still not fully understood. However, it is believed that the presence of lipofuscin can contribute to cellular dysfunction, oxidative damage, and impaired proteostasis, all of which are key features of aging and age-related diseases. Increased lipofuscin accumulation has been associated with impairment of autophagy and mitophagy, two critical cellular processes involved in the degradation and removal of damaged or dysfunctional cellular components.

Autophagy is a cellular recycling process that involves the sequestration and degradation of cytoplasmic components, including damaged proteins and organelles, within specialized double-membrane structures called autophagosomes. Lipofuscin accumulation can disrupt autophagy through several mechanisms. First, by reduction of sequestration of autophagic components. Lipofuscin reduces the number of active lysosomes, where the degradation of autophagosomes takes place. This physical obstruction hinders the fusion between autophagosomes and lysosomes, preventing the efficient clearance of cellular waste [132]. Second, by lysosomal dysfunction. Lipofuscin-filled lysosomes exhibit greatly impaired enzymatic activity and altered pH, compromising their ability to degrade cargo delivered by autophagosomes. The altered lysosomal environment hinders the activity of lysosomal hydrolases, thus impairing autophagic degradation [132]. Third by disruption of autophagic signaling. Lipofuscin accumulation disrupts the signaling pathways involved in the initiation and regulation of autophagy. This interference can lead to the dysregulation of key autophagy-related proteins and transcription factors, resulting in impaired autophagic flux [132,133].

Mitophagy is a specific form of autophagy that targets damaged or dysfunctional mitochondria for degradation. Healthy mitochondria are crucial for cellular energy production and the maintenance of cellular homeostasis. Lipofuscin accumulation can interfere with mitophagy in the following ways. Impaired Mitochondrial Turnover: Lipofuscin-filled lysosomes can hinder the efficient removal of damaged mitochondria through mitophagy. The impaired fusion between autophagosomes and lysosomes prevents the degradation of dysfunctional mitochondria, leading to their accumulation. Second, Oxidative Damage to Mitochondria: Lipofuscin accumulation is associated with oxidative stress, and oxidative damage can directly affect mitochondrial function and integrity. Damaged mitochondria may become less recognizable or accessible to mitophagy receptors, impairing the recognition and targeting of damaged mitochondria for degradation. Third, Disruption of Mitophagy Signaling: Lipofuscin accumulation can interfere with the signaling pathways involved in the initiation and regulation of mitophagy. Dysregulation of key mitophagy-related proteins and transcription factors may result in impaired clearance of damaged mitochondria [134,135].

## Mitochondrial dysfunction in common neurodegenerative disorders: recent advances in therapeutics

### Mitochondrial dysfunction in Alzheimer's disease

Dementia is a group of symptoms including difficulties with memory, language, problem-solving, and other thinking skills [136]. The most common cause of dementia is Alzheimer's disease (AD), which is associated with 60–80 ​% of all cases [136]. AD is characterized by the accumulation of extracellular beta-amyloid (Aβ) and intracellular tau protein neurofibrillary tangles [136,137]. Altered mitochondrial morphology and function have also been observed in AD, however, the temporality of association is debated [137]. Competing hypotheses of AD progression include the amyloid cascade hypothesis, where Aβ and tau accumulation drive mitochondrial dysfunction, and the mitochondrial cascade hypothesis, which posits that Aβ deposition occurs secondary to mitochondrial dysfunction, which is the initial trigger of AD [137,138]. In early-onset familial AD, protein accumulation is likely to be the first step, however, in the more common late-onset sporadic form the debate continues [139]. In both hypotheses, mitochondrial dysfunction plays a central role in the etiology of AD (Fig. 2).

Both hypotheses of AD pathogenesis hold some validity because mitochondrial dysfunction can be driven not only by the mechanisms discussed earlier but also by AD-specific proteins such as Aβ oligomers. Aβ and Aβ precursor protein (APP) disrupt mitochondrial function by altering typical mitochondrial physiology. One way this occurs is through Aβ interaction with complexes within the mitochondria, including Aβ-binding alcohol dehydrogenase and cyclophilin D [138]. Additionally, APP dysfunction reduces cytochrome oxidase activity [138]. Importantly, Aβ has also been found to disrupt intracellular calcium homeostasis, leading to increased cell membrane excitability and neuronal dysfunction [138,140].

Independent of Aβ involvement, inherited mtDNA variations may influence vulnerability to AD, however, it is likely that they are not sufficient to cause AD on their own [141]. Additionally, ROS play a role in the etiology and/or progression of AD. In the brain of AD patients, mtDNA has been found to have 10-fold higher levels of oxidized bases than nuclear DNA and, when compared to age-matched controls, AD patients had a 3-fold increase in oxidative damage in the brain [141].

These disturbances in mitochondrial function cause disruptions in typical function that may drive AD progression [141]. Abnormal mitochondria fission and fusion, abnormal mitochondrial distribution throughout pyramidal neurons, failing mitochondrial biogenesis, mitochondria-associated membrane dysfunction, inadequate mitophagy, and impaired proteostasis are all hallmarks of mitochondrial dysfunction that contribute to the progression of AD [141]. Many of these abnormalities may be therapeutically preventable either through directly targeting the mitochondria, such as with Drp1 inhibitors, or through targeting Aβ or tau to inhibit any interaction with the mitochondria [141,142]. Many of the current clinical trials for treatments to prevent AD or slow AD progression focus on the latter method, or on introducing antioxidants to prevent ROS from damaging mitochondria and other cellular structures.

#### Current clinical trials

Nicotinamide and MIB-626 (a nicotinamide precursor) are potential therapeutics with several ongoing clinical trials listed in Table 1. The proposed method of action for nicotinamide is through the conversion of nicotinamide to NAD^+^, inhibiting sirtuin and reducing levels of Thr231-Phosphotau [143]. Orally administered nicotinamide was shown to mitigate Aβ and tau pathologies and improve cognitive function in mice [144].

**Table 1:** Current clinical trials targeting mitochondrial dysfunction in neurodegenerative diseases.

ID	Treatment	Phase	N
*Alzheimer's disease*			
NCT05617508	NR	N/A	80
NCT04430517	NR	I	50
NCT05040321	MIB-626	I/II	50
NCT04842552	Hydralazine	III	424
NCT05591027	*Centella asiatica* product	I	48
NCT04740580	Glycine, NAC	I	52
NCT05081219	Insulin, empagliflozin	II	60
NCT04018092	Photobiomodulation	II	168
NCT04784416	Photobiomodulation	II	125
*Amyotrophic lateral sclerosis*			
NCT04820478	Beta hydroxybutyrate ester	N/A	76
NCT04244630	Antioxidant supplements	II	60
*Huntington's disease*			
NCT01502046	THC and CBD	II	25
*Parkinson's disease*			
NCT04477161	Oral Ketone Esters	N/A	10
NCT03840005	Ursodeoxycholic acid	II	31
NCT04044131	Serine, l-carnitine, NAC, NR	II	120
NCT05214287	Intermittent hypoxia therapy	I, II	29
NCT04768023	Vitamin D3	N/A	50

A current list of ongoing clinical trials ascertained from ClinicalTrials.gov. The following search terms were used: Alzheimer's Disease, Amyotrophic lateral sclerosis, ALS, Lou Gehrig's, Huntington's Disease, Parkinson's Disease, Parkinsonism, mitochondria, mitochondrial, bioenergetics. Abbreviations: N – number of participants, NR – nicotinamide riboside, NAC – N-acetylcysteine, THC – Tetrahydrocannabinol, CBD – Cannabidiol.

Another drug currently in clinical trial is hydralazine. Hydralazine is an FDA-approved drug for hypertension, however, recent research suggests it may also be useful for delaying or preventing oxidative stress-related disorders such as Alzheimer's [145]. This drug activates the nuclear factor erythroid-derived 2-related factor (Nrf2) pathway, leading to antioxidant gene transcription and prevention of oxidative stress [145]. Additionally, it may be useful as a scaffold molecule to prevent misfolding of Aβ [146]. An extract of *Centella asiatica* similarly activates the Nrf2 pathway and is the focus of a separate clinical trial [147].

Glutathione is a molecule involved with the regulation of homeostasis and metabolism in the brain [148]. Deficiency or impairment of glutathione can lead to loss of neurons in the brain due to glutathione's important role as an antioxidant, among other things [148]. One phase I clinical trial is attempting to supplement participants' diets with glycine and N-acetylcysteine, glutathione precursors.

Due to impaired insulin metabolism in the brain in AD, nasally administered insulin and empagliflozin are also being examined [149] In the past, there have been varied results with intranasal insulin – some studies have shown improved memory and cognitive function while others have shown no improvement [149].

The only non-drug therapeutic currently undergoing clinical trial is transcranial photobiomodulation. Photobiomodulation involves applying electromagnetic radiation in the visible light range and near infrared that may activate NADH dehydrogenase, cytochrome *c* reductase, and cytochrome *c* oxidase [150]. It has been shown to reduce Aβ in vitro [151].

### Mitochondrial dysfunction in amyotrophic lateral sclerosis

Amyotrophic lateral sclerosis (ALS), sometimes referred to as Lou Gehrig's disease, is the most common type of motor neuron disease in adults [152]. Some 5–10 ​% of all ALS cases are familial; the remaining 90–95 ​% of cases are sporadic and idiopathic [152]. Mitochondrial dysfunction in ALS is often caused by protein aggregates and mutations to mitochondrial protein components (Fig. 3) [153]. Mutations in genes such as superoxide dismutase 1 (*SOD1*) and TAR DNA-binding protein 43 (*TDP-43*) impair mitochondrial calcium uptake [154]. These mutations can also lead to proteinopathy and intensified mitochondrial dysfunction. Aggregates of TDP-43 are typically found in the neuronal cytoplasm of ALS patients and this aggregation impairs regulation of mitochondrial transcripts [154]. There is some evidence that TDP-43 may be a prion-like self-propagating protein aggregate such as Aβ and tau [153].

The coiled-coil-helix-coiled-coil-helix domain containing 10 (CHCHD10) mitochondrial protein is important in the formation and maintenance of cristae junctions [155]. Within the mitochondria, this protein also interacts with CHCHD2, creating a complex that is required for efficient mitochondrial respiration [155] One mutation of *CHCHD10* has been shown to lead to the fragmentation of the mitochondrial network and the loss of cristae junctions [155]. Under mitochondrial stress, CHCHD10 moves from the mitochondria to the cytoplasm to directly interact with TDP-43, moving TDP-43 back into the nucleus to prevent the protein aggregates [155]. Mutations in *CHCHD10* may cause this translocation to be less efficient [155].

The *SOD1* gene, identified as a cause of ALS 30 years ago, was foundational in the study of ALS pathogenesis [156]. There are many known *SOD1* mutations, and they are responsible for 12 ​% of the incidence of familial ALS and 1–2% of the incidence of sporadic ALS [153,156] It should be noted that SOD1 ALS lacks the TDP-43 pathology that is present in nearly every other ALS case. Because of this, many consider SOD1 ALS a distinct form of the disease [156].

Another landmark event for ALS research was the discovery of a mutation in the noncoding region of the *C9orf72* gene [155]. The haploinsufficiency of this gene is implicated in about 40 ​% of familial cases of ALS as well as many sporadic cases and provides a genetic link between the pathogenesis of ALS and frontotemporal dementia [157,158]. We are only beginning to understand the role of C9orf72 in the cell, but recent advances show that it is a MIM-associated protein that plays a critical role in oxidative phosphorylation [157]. The loss of C9orf72 appears to be a sufficient cause of cell death in motor neurons under the conditions of energetic stresses [157].

#### New treatments and current clinical trials

The current clinical trials examining ALS therapeutics targeting mitochondria are listed in Table 1. There are two dietary intervention clinical trials currently ongoing. One of the studies examines the efficacy and tolerability of β-hydroxybutyrate ester, a ketone body dietary supplement, and the other studies an antioxidant supplement.

A new therapeutic, tofersen, was recently approved by the U.S. Food and Drug Administration for the treatment of *SOD1* ALS [159,160]. Tofersen is an antisense oligonucleotide. Because antisense oligonucleotides are unable to cross the blood-brain barrier, they must be injected intrathecally [153,160]. Once administered, antisense oligonucleotides reduce protein expression through either forming a duplex with target pre-mRNA or mRNA, leading to ribonuclease H degradation, or through interfering with target pre-mRNA or mRNA translation and/or splicing [153]. A collection of clinical trials examining antisense oligonucleotides for the treatment of ALS are listed in Ref. [153]. An antisense oligonucleotide targeting C9orf72 ALS, WVE-004, showed promise in preclinical examination, however, development was recently canceled after a phase I/II clinical trial failed to show benefit compared to a placebo [161,162].

### Mitochondrial dysfunction in Huntington's disease

Huntington's disease (HD) is a neurodegenerative movement disorder characterized by degeneration of the caudate nucleus and the putamen [163]. Specific loss of efferent medium spiny neurons is also common with HD. These cause significant symptoms including motor defects such as chorea and coordination loss as well as psychiatric symptoms such as depression and psychosis. HD onset is due to a mutation in the *HTT* gene, which codes for huntingtin. This mutation is a dominantly inherited CAG repeat expansion within exon 1 of the *HTT* gene [164]. Huntingtin is a protein with several important physiological functions including neurogenesis in the embryo, acting as a scaffolding protein (including localization to spindle poles during mitosis), regulating transcription (namely in the gene that codes for brain-derived neurotrophic factor), and it plays a role in the correct formation of synapses [163]. The presence of HTT in embryonic stage is extremely important as mice with the *HTT* gene knocked out are reported to die before birth [164]. Mutation in huntingtin can lead to transcriptional dysfunction and damage to the mitochondria [165]. This mitochondrial damage has a cascading effect on HD causing excitotoxic stress, increased expression of inflammatory signals, oxidative injury, and pro-apoptotic signals (Fig. 4). All these cascading effects lead to further neuronal damage and eventual death that is observed in HD. Mitochondrial dysfunction is especially drastic in neuronal cells as these cells require exceptional metabolic capabilities to function properly. Huntingtin dysfunction is also hypothesized to lead to improper mitochondrial fusion and fission [166]. This is caused by huntingtin potentially influencing the development of key mobility proteins, causing mitochondria to have impaired trafficking and mobility.

In addition to other suggested means of pathogenesis of HD, deficiency in heat shock transcription factor 1 (HSF1) could also be involved [167]. HSF1 has roles in both the regulation of gene expression within the nucleus and the regulation of oxidative stress within the mitochondria, the means by which this is accomplished is currently unknown [167]. Because of this, if HSF1 is missing or functions improperly, mitochondrial function is heavily affected, as a deficit in HSF1 in the nucleus leads to improper gene expression. HSF1 deficiency induces oxidative stress that is already prevalent in HD as well as mitochondrial biogenesis inhibition. Studies have shown that HSF1 accumulation within the mitochondria results in both neurodegeneration and mitochondrial movement deficits. Therefore, the idea of inhibiting HSF1 in the mitochondria to some extent as a potential therapeutic option comes to fruition. However, inhibiting HSF1 nonselectively can lead to further detrimental effects and thus is not a viable option. However, other means of accomplishing this are being studied. One example is either blocking or inhibiting the association that exists between HSF1 and mitochondria. This was accomplished by introducing DH1, a unique peptide inhibitor that blocks the involvement of HSF1 in mitochondria [167]. This results in an increase in mitochondria elongation and an increase in mitochondrial nucleoids, implying that DH1 treatment improves the replication of mtDNA. The introduction of DH1 allows for slower neurodegeneration and the restoration of mitochondrial function through competition with HSF1 for dynamin-related protein 1 (Drp1) binding. When evoked by HSF1, Drp1 will begin phosphorylation, inducing shortening of the mitochondria, returning them to their normal shape. However, further studies still need to be performed in order to determine any other potential effects this may have, as well as determine the best means of administration of the treatment. The use of osmotic pumps in mice demonstrated that DH1 was found in the cortex and striatum, which suggests that DH1 has the capability to cross the blood-brain barrier [167]. The treatment with DH1 improved both neurotoxicity and behavior in an animal model. Further studies are being conducted on the effects of this treatment on human cerebral organoids, as the cross-species effects of this treatment are not well understood currently.

### Mitochondrial dysfunction in Parkinson's disease

Parkinson's disease (PD) is a neurodegenerative disease characterized by key motor symptoms that include hypokinesia, bradykinesia, resting tremor, postural instability, and rigidity [168]. Other non-motor symptoms are noted in PD as well, including dyssomnias, depression, dementia, and autonomic dysfunction. These symptoms typically precede the motor symptoms, but this is not the case consistently. The motor symptoms are brought on by degeneration of the dopaminergic (DA) neurons, specifically within the substantia nigra pars compacta (SNc). This degeneration results in a notable lack of dopamine within the synapses and deranged neuronal circuits in the target areas of dopamine, such as the basal ganglia.

Many means of pathogenesis of PD exist. One of the most heavily studied is Lewy bodies [169]. Mitochondrial dysfunction is also heavily prevalent in PD [169]. There are several ways in which mitochondrial dysfunction may contribute to the pathogenesis of PD, most notably through a decrease in the activity of mitochondrial complex enzyme I (Fig. 5). The decrease in activity leads to additional oxidative stress on nigral neurons, thus harming the integrity of the neuron, and leading to neurodegeneration. Risk factors of PD include aging, namely due to the increased failure of cellular compensatory mechanisms in the brain as aging progresses, and environmental factors such as toxins located in the environment including pesticides that inhibit complex-I in the mitochondria or other toxins that can alter mitochondrial gene expression and protein function.

Genetic factors play a substantial role in the pathogenesis of PD, and there is a plethora of genes that are or could be involved. These genes include *SNCA*, *PARK2*, *UCHL1*, *PINK1*, *DJ-1*, *LRRK2*, *ATP13A2*, and *HTRA2*. Mutations and dysfunctions in these genes lead to a wide range of effects including a decrease in complex I activity, decreased integrity of the mitochondria, decreased ATP production, general disruption of the morphology of the mitochondria, UPS (ubiquitin-proteasomal system) dysfunction, and ALP (autophagy lysosomal pathway) dysfunction. More specifically, *SNCA*, or the α-synuclein gene, codes for a protein that is an element of Lewy bodies [170]. Accumulation of α-synuclein within the mitochondria will interfere with complex I, thus increasing mitophagy. Through this method, excess calcium can trigger mitochondrial dysfunction via the α-synuclein pathway. Mutations in the mitochondria-specific kinase PTEN Induced Kinase 1 (PINK1) also share the Lewy body pathology as well as specific loss of DA neurons [171]. PINK1 plays a large role in the repair of mitochondrial dysfunction as it will respond to damage to individual mitochondria. This is accomplished by recruiting parkin and triggering mitochondrial autophagy. Thus, novel methods are being studied to restore PINK1 function in patients with PD, though the majority of the studies are in the early stages [172].

Currently, PD is screened via biomarkers such as α-synuclein levels and isoforms in blood, genetic screening, and mitochondrial complex I measurement [169]. However, with the knowledge of mitochondrial dysfunction in PD, other biomarkers may be present and allow for earlier detection of the disease. Therapeutic development for PD is also further enabled by an understanding of mitochondrial dysfunction, as targeting the dysfunction is another means to solve the issue. This is notably important because currently the only treatments presented to patients with PD are primarily used to treat the symptoms, not the disease [173]. These treatments have a primary purpose of increasing the levels of dopamine within the brain, namely areas of the midbrain. However, these have no neuroprotective effects. One proposed therapeutic is creatine supplementation, as this has been shown to have a neuroprotective effect in mouse models [169]. However, creatine supplementation as a therapeutic within a human model is still debated over the efficacy of the solution, as the supplementation had little to no effect on the Unified Parkinson's Disease Rating Scale scores [169,174]. Another proposed therapeutic is coenzyme Q10 administration. Coenzyme Q10 is a lipid-soluble compound that acts as an electron carrier in the electron transport chain. When reduced, the compound forms ubiquinol, which is an antioxidant located in the membranes of mitochondria. The efficacy of treatment with coenzyme Q10 is still debated but protective effects of the treatment were noted in DA neurons within a mouse model [169,175]. Another supplementary option is vitamin K2, as it was able to rescue motor disturbances in Drosophila but failed to have any notable effects in mammalian cells.

## Future directions

Neurodegenerative disorders were initially believed to be disorders of protein homeostasis and characterized by the presence of aberrant protein accumulation, specifically α-synuclein, tau, and amyloid protein aggregation and deposition. However, recent research studies that specifically probed into familial variants of several neurodegenerative disorders have identified numerous additional processes that may significantly contribute to the pathogenesis of these complex clinically heterogeneous conditions. The mechanisms encompass a wide range of factors, including perturbations in proteostasis and lysosomal trafficking, impairment of mitochondrial function, increased oxidative stress, and dysregulation of calcium and iron homeostasis. Oxidative stress and mitochondrial dysfunction are well recognized as fundamental pathophysiological factors underlying the majority of neurodegenerative diseases [[176], [177], [178]]. These mechanistic insights have resulted in a change in the focus of research conducted in the last decade, with the aim of identifying biomarkers associated with oxidative stress and mitochondrial dysfunction in neurodegenerative illnesses characterized by unique phenotypes, including Alzheimer's disease (AD), Parkinson's disease (PD), and amyotrophic lateral sclerosis (ALS). Moreover, the role of oxidative stress due to impaired redox homeostasis resulting in the progressive loss of different subtypes of neurons has been well established, which is further supported by the findings of elevated levels of oxidized byproducts derived from lipids, proteins, and nucleic acids in postmortem examinations of brain tissues from individuals afflicted with neurodegenerative disorders [179,180]. There is increasing evidence suggesting that mitochondrial dysfunction plays a significant role in the etiopathogenesis and progression of neurodegenerative illnesses. Additionally, an imbalanced redox state may contribute to disruptions in proteostasis, leading to the accumulation of toxic protein aggregates such as Lewy bodies and neurofibrillary tangles [170,181,182].

Despite the progress in understanding various pathomechanisms contributing to the development and progression of neurodegenerative disorders, no definitive treatment option is available to delay the progression of the disease. Hence, strategies aimed at modulating oxidative dysfunction and redox stress are promising options for modifying the trajectory of neurodegenerative diseases [183]. Several potential targets have been considered, including techniques aimed at replenishing NAD ​+ ​reserves, antioxidants such as CoQ10, and modulators of NRF2 [183]. The association between elevated levels of Protein inhibitor of activated STAT2 (PIAS2) and neurodegeneration has been shown, because of its interaction with the STAT and p53 pathways. Consequently, there is ongoing research and interest in the therapeutic potential of modulating PIAS2 [184]. Cellular energy demands dictate a delicate equilibrium between the biogenesis and degradation of mitochondria. This intricate interplay is governed by nuclear respiratory factors, namely NRF1 and NRF2, working with multiple transcription regulators that span both the mitochondrial and nuclear genomes [185,186]. This has led to an ongoing pursuit of NRF upregulation in various clinical trials aimed at disease-modifying strategies for individuals with neurodegenerative disorders [[187], [188], [189]].

Using multipronged approaches aimed at reducing the generation of reactive oxygen species (ROS) and regulating the processes of mitochondrial biogenesis and homeostasis is increasingly recognized as a cohesive principle in the treatment of neurodegenerative diseases. Nevertheless, these strategies' effectiveness will rely on the identification of biomarkers associated with mitochondrial dysfunction. The primary objective will be to detect the onset of neurodegeneration in its prodromal or preclinical phases, thereby improving the therapeutic outcomes. The clinical application of omics technology has enabled the potential for utilizing molecular signatures to stratify individuals with neurodegenerative illnesses [190]. Additionally, these omics techniques could be employed to predict the ex vivo effectiveness of mitochondrial modulation therapy.

## Declaration of competing interest

The authors declare that they have no known competing financial interests or personal relationships that could have appeared to influence the work reported in this paper.

## References

[1] Ballard J.W., Whitlock M.C. (2004). The incomplete natural history of mitochondria. Mol Ecol.

[2] Di Donato S. (2000). Disorders related to mitochondrial membranes: pathology of the respiratory chain and neurodegeneration. J Inherit Metab Dis.

[3] Klecker T., Westermann B. (2021). Pathways shaping the mitochondrial inner membrane. Open Biol.

[4] Kramer P., Bressan P. (2018). Our (Mother's) mitochondria and our mind. Perspect Psychol Sci.

[5] Frey T.G., Mannella C.A. (2000). The internal structure of mitochondria. Trends Biochem Sci.

[6] Zeviani M., Simonati A., Bindoff L.A., Subramony S.H., Dürr A. (2012).

[7] Haas R.H. (2007). The evidence basis for coenzyme Q therapy in oxidative phosphorylation disease. Mitochondrion.

[8] Neupert W., Herrmann J.M. (2007). Translocation of proteins into mitochondria. Annu Rev Biochem.

[9] Chinnery P.F., Elliott H.R., Hudson G., Samuels D.C., Relton C.L. (2012). Epigenetics, epidemiology and mitochondrial DNA diseases. Int J Epidemiol.

[10] Chacinska A., Pfannschmidt S., Wiedemann N., Kozjak V., Sanjuán Szklarz L.K., Schulze-Specking A. (2004). Essential role of Mia40 in import and assembly of mitochondrial intermembrane space proteins. EMBO J.

[11] Naoé M., Ohwa Y., Ishikawa D., Ohshima C., Nishikawa S., Yamamoto H. (2004). Identification of Tim40 that mediates protein sorting to the mitochondrial intermembrane space. J Biol Chem.

[12] Mannella C.A. (2006). Structure and dynamics of the mitochondrial inner membrane cristae. Biochim Biophys Acta.

[13] Vogel F., Bornhövd C., Neupert W., Reichert A.S. (2006). Dynamic subcompartmentalization of the mitochondrial inner membrane. J Cell Biol.

[14] van der Bliek A.M., Sedensky M.M., Morgan P.G. (2017). Cell Biology of the Mitochondrion. Genetics.

[15] Luft R., Ikkos D., Palmieri G., Ernster L., Afzelius B. (1962). A case of severe hypermetabolism of nonthyroid origin with a defect in the maintenance of mitochondrial respiratory control: a correlated clinical, biochemical, and morphological study. J Clin Investig.

[16] Taylor R.W., Pyle A., Griffin H., Blakely E.L., Duff J., He L. (2014). Use of whole-exome sequencing to determine the genetic basis of multiple mitochondrial respiratory chain complex deficiencies. JAMA.

[17] Anderson S., Bankier A.T., Barrell B.G., de Bruijn M.H.L., Coulson A.R., Drouin J. (1981). Sequence and organization of the human mitochondrial genome. Nature.

[18] McFarland R., Taylor R.W., Turnbull D.M. (2010). A neurological perspective on mitochondrial disease. Lancet Neurol.

[19] Attwell D., Laughlin S.B. (2001). An energy budget for signaling in the grey matter of the brain. J Cereb Blood Flow Metab.

[20] Coyle J.T., Puttfarcken P. (1993). Oxidative stress, glutamate, and neurodegenerative disorders. Science.

[21] Gould E. (2007). How widespread is adult neurogenesis in mammals?. Nat Rev Neurosci.

[22] Sheng Z.-H., Cai Q. (2012). Mitochondrial transport in neurons: impact on synaptic homeostasis and neurodegeneration. Nature Reviews Neuroscience.

[23] Harman D. (1972). The biologic clock: The mitochondria?. J Am Geriatr Soc.

[24] Langston J.W., Ballard P., Tetrud J.W., Irwin I. (1983). Chronic Parkinsonism in humans due to a product of meperidine-analog synthesis. Science.

[25] Ramsay R.R., Dadgar J., Trevor A., Singer T.P. (1986). Energy-driven uptake of N-methyl-4-phenylpyridine by brain mitochondria mediates the neurotoxicity of MPTP. Life Sci.

[26] Ramsay R.R., Salach J.I., Dadgar J., Singer T.P. (1986). Inhibition of mitochondrial NADH dehydrogenase by pyridine derivatives and its possible relation to experimental and idiopathic parkinsonism. Biochem Biophys Research Commun.

[27] Greenamyre J.T., Sherer T.B., Betarbet R., Panov A.V. (2001). Complex I and Parkinson's disease. IUBMB Life.

[28] Sherer T.B., Betarbet R., Stout A.K., Lund S., Baptista M., Panov A.V. (2002). An in vitro model of Parkinson's disease: Linking mitochondrial impairment to altered α-synuclein metabolism and oxidative damage. J Neurosci.

[29] Lannuzel A., Michel P.P., Höglinger G.U., Champy P., Jousset A., Medja F. (2003). The mitochondrial complex i inhibitor annonacin is toxic to mesencephalic dopaminergic neurons by impairment of energy metabolism. Neuroscience.

[30] Bindoff L.A., Birch-Machin M., Cartlidge N.E.F., Parker W.D.J., Turnbull D.M. (1989). Mitochondrial function in Parkinson's disease. Lancet.

[31] Schapira A.H.V., Cooper J.M., Dexter D., Clark J.B., Jenner P., Marsden C.D. (1990). Mitochondrial complex I deficiency in Parkinson's disease. J Neurochem.

[32] Schapira A.H.V., Cooper J.M., Dexter D., Jenner P., Clark J.B., Marsden C.D. (1989). Mitochondrial complex I deficiency in Parkinson's disease. Lancet.

[33] Przedborski S., Jackson-Lewis V., Fahn S. (1995). Antiparkinsonian therapies and brain mitochondrial complex I activity. Mov Disord.

[34] Saraiva A.A., Borges M.M., Madeira M.D., Tavares M.A., Paula-Barbosa M.M. (1985). Mitochondrial abnormalities in cortical dendrites from patients with Alzheimer's disease. J Submicrosc Cytol.

[35] Sumpter P.Q., Mann D.M., Davies C.A., Yates P.O., Snowden J.S., Neary D. (1986). An ultrastructural analysis of the effects of accumulation of neurofibrillary tangle in pyramidal neurons of the cerebral cortex in Alzheimer's disease. Neuropathol Appl Neurobiol.

[36] Peterson C., Goldman J.E. (1986). Alterations in calcium content and biochemical processes in cultured skin fibroblasts from aged and Alzheimer donors. Proc Natl Acad Sci U S A.

[37] Swerdlow R.H., Koppel S., Weidling I., Hayley C., Ji Y., Wilkins H.M. (2017). Mitochondria, cybrids, aging, and Alzheimer's disease. Prog Mol Biol Transl Sci.

[38] Cadonic C., Sabbir M.G., Albensi B.C. (2016). Mechanisms of mitochondrial dysfunction in Alzheimer's disease. Mol Neurobiol.

[39] Parker W.D., Filley C.M., Parks J.K. (1990). Cytochrome oxidase deficiency in Alzheimer's disease. Neurology.

[40] Parker W.D., Mahr N.J., Filley C.M., Parks J.K., Hughes D., Young D.A. (1994). Reduced platelet cytochrome c oxidase activity in Alzheimer's disease. Neurology.

[41] Kish S.J., Mastrogiacomo F., Guttman M., Furukawa Y., Taanman J.W., Dozić S. (1999). Decreased brain protein levels of cytochrome oxidase subunits in Alzheimer's disease and in hereditary spinocerebellar ataxia disorders: a nonspecific change?. J Neurochem.

[42] Kaminsky Y.G., Tikhonova L.A., Kosenko E.A. (2015). Critical analysis of Alzheimer's amyloid-beta toxicity to mitochondria. Front Biosci (Landmark Ed)..

[43] Engelender S., Sharp A.H., Colomer V., Tokito M.K., Lanahan A., Worley P. (1997). Huntingtin-associated protein 1 (HAP1) interacts with the p150Glued subunit of dynactin. Hum Mol Genet.

[44] Borthwick G.M., Johnson M.A., Ince P.G., Shaw P.J., Turnbull D.M. (1999). Mitochondrial enzyme activity in amyotrophic lateral sclerosis: implications for the role of mitochondria in neuronal cell death. Ann Neurol.

[45] Liu J., Lillo C., Jonsson P.A., Vande Velde C., Ward C.M., Miller T.M. (2004). Toxicity of familial ALS-linked SOD1 mutants from selective recruitment to spinal mitochondria. Neuron.

[46] Sturtz L.A., Diekert K., Jensen L.T., Lill R., Culotta V.C. (2001). A fraction of yeast Cu,Zn-superoxide dismutase and its metallochaperone, CCS, localize to the intermembrane space of mitochondria. A physiological role for SOD1 in guarding against mitochondrial oxidative damage. J Biol Chem.

[47] Wright A.F., Jacobson S.G., Cideciyan A.V., Roman A.J., Shu X., Vlachantoni D. (2004). Lifespan and mitochondrial control of neurodegeneration. Nat Genet.

[48] Choo Y.S., Johnson G.V., MacDonald M., Detloff P.J., Lesort M. (2004). Mutant huntingtin directly increases susceptibility of mitochondria to the calcium-induced permeability transition and cytochrome c release. Hum Mol Genet.

[49] Bender A., Krishnan K.J., Morris C.M., Taylor G.A., Reeve A.K., Perry R.H. (2006). High levels of mitochondrial DNA deletions in substantia nigra neurons in aging and Parkinson disease. Nature Genetics.

[50] Carelli V., Achilli A., Valentino M.L., Rengo C., Semino O., Pala M. (2006). Haplogroup effects and recombination of mitochondrial DNA: novel clues from the analysis of Leber hereditary optic neuropathy pedigrees. Am J Hum Genet.

[51] Hardy J., Cai H., Cookson M.R., Gwinn-Hardy K., Singleton A. (2006). Genetics of Parkinson's disease and parkinsonism. Ann Neurol.

[52] Davidzon G., Greene P., Mancuso M., Klos K.J., Ahlskog J.E., Hirano M. (2006). Early-onset familial parkinsonism due to POLG mutations. Ann Neurol.

[53] Devi L., Prabhu B.M., Galati D.F., Avadhani N.G., Anandatheerthavarada H.K. (2006). Accumulation of amyloid precursor protein in the mitochondrial import channels of human Alzheimer's disease brain is associated with mitochondrial dysfunction. J Neurosci.

[54] Greenamyre J.T. (2007). Huntington's disease--making connections. N Engl J Med.

[55] DiMauro S., Quinzii C.M., Hirano M. (2007). Mutations in coenzyme Q10 biosynthetic genes. J Clin Invest.

[56] DiMauro S., Schon E.A. (2003). Mitochondrial respiratory-chain diseases. N Engl J Med.

[57] DiMauro S., Schon E.A., Carelli V., Hirano M. (2013). The clinical maze of mitochondrial neurology. Nat Rev Neurol.

[58] Acin-Perez R., Enriquez J.A. (2014). The function of the respiratory supercomplexes: the plasticity model. Biochim Biophys Acta.

[59] Genova M.L., Lenaz G. (2014). Functional role of mitochondrial respiratory supercomplexes. Biochim Biophys Acta.

[60] Koopman W.J., Nijtmans L.G., Dieteren C.E., Roestenberg P., Valsecchi F., Smeitink J.A. (2010). Mammalian mitochondrial complex I: biogenesis, regulation, and reactive oxygen species generation. Antioxid Redox Signal.

[61] Efremov R.G., Baradaran R., Sazanov L.A. (2010). The architecture of respiratory complex I. Nature.

[62] Cecchini G. (2003). Function and structure of complex II of the respiratory chain. Annu Rev Biochem.

[63] Tsukihara T., Aoyama H., Yamashita E., Tomizaki T., Yamaguchi H., Shinzawa-Itoh K. (1996). The whole structure of the 13-subunit oxidized cytochrome c oxidase at 2.8 A. Science.

[64] Ferguson S.J. (2000). ATP synthase: what dictates the size of a ring?. Curr Biol.

[65] Yoshida M., Muneyuki E., Hisabori T. (2001). ATP synthase--a marvellous rotary engine of the cell. Nat Rev Mol Cell Biol.

[66] Liu Y., Levine B. (2015). Autosis and autophagic cell death: the dark side of autophagy. Cell Death Differ.

[67] Jeong S.Y., Seol D.W. (2008). The role of mitochondria in apoptosis. BMB Rep.

[68] Pradelli L.A., Bénéteau M., Ricci J.E. (2010). Mitochondrial control of caspase-dependent and -independent cell death. Cell Mol Life Sci.

[69] Orrenius S., Gogvadze V., Zhivotovsky B. (2015). Calcium and mitochondria in the regulation of cell death. Biochem Biophys Res Commun.

[70] Morciano G., Giorgi C., Bonora M., Punzetti S., Pavasini R., Wieckowski M.R. (2015). Molecular identity of the mitochondrial permeability transition pore and its role in ischemia-reperfusion injury. J Mol Cell Cardiol.

[71] Morciano G., Giorgi C., Balestra D., Marchi S., Perrone D., Pinotti M. (2016). Mcl-1 involvement in mitochondrial dynamics is associated with apoptotic cell death. Mol Biol Cell.

[72] Bonora M., Pinton P. (2014). The mitochondrial permeability transition pore and cancer: molecular mechanisms involved in cell death. Front Oncol.

[73] Vakifahmetoglu-Norberg H., Ouchida A.T., Norberg E. (2017). The role of mitochondria in metabolism and cell death. Biochem Biophys Res Commun.

[74] Fatokun A.A., Dawson V.L., Dawson T.M. (2014). Parthanatos: mitochondrial-linked mechanisms and therapeutic opportunities. Br J Pharmacol.

[75] Virág L., Robaszkiewicz A., Rodriguez-Vargas J.M., Oliver F.J. (2013). Poly(ADP-ribose) signaling in cell death. Mol Aspects Med.

[76] Andrabi S.A., Dawson T.M., Dawson V.L. (2008). Mitochondrial and nuclear cross talk in cell death: parthanatos. Ann N Y Acad Sci.

[77] Angelova P.R., Abramov A.Y. (2018). Role of mitochondrial ROS in the brain: from physiology to neurodegeneration. FEBS Lett.

[78] Patron M., Raffaello A., Granatiero V., Tosatto A., Merli G., De Stefani D. (2013). The mitochondrial calcium uniporter (MCU): molecular identity and physiological roles. J Biol Chem.

[79] Kamer K.J., Mootha V.K. (2014). MICU1 and MICU2 play nonredundant roles in the regulation of the mitochondrial calcium uniporter. EMBO Rep.

[80] Sancak Y., Markhard A.L., Kitami T., Kovács-Bogdán E., Kamer K.J., Udeshi N.D. (2013). EMRE is an essential component of the mitochondrial calcium uniporter complex. Science.

[81] Kamer K.J., Mootha V.K. (2015). The molecular era of the mitochondrial calcium uniporter. Nat Rev Mol Cell Biol.

[82] Szymański J., Janikiewicz J., Michalska B., Patalas-Krawczyk P., Perrone M., Ziółkowski W. (2017). Interaction of Mitochondria with the Endoplasmic Reticulum and Plasma Membrane in Calcium Homeostasis, Lipid Trafficking and Mitochondrial Structure. Int J Mol Sci.

[83] Palty R., Sekler I. (2012). The mitochondrial Na(+)/Ca(2+) exchanger. Cell Calcium.

[84] Gincel D., Zaid H., Shoshan-Barmatz V. (2001). Calcium binding and translocation by the voltage-dependent anion channel: a possible regulatory mechanism in mitochondrial function. Biochem J.

[85] Keinan N., Pahima H., Ben-Hail D., Shoshan-Barmatz V. (2013). The role of calcium in VDAC1 oligomerization and mitochondria-mediated apoptosis. Biochim Biophys Acta.

[86] Weisthal S., Keinan N., Ben-Hail D., Arif T., Shoshan-Barmatz V. (2014). Ca(2+)-mediated regulation of VDAC1 expression levels is associated with cell death induction. Biochim Biophys Acta.

[87] Clapham D.E. (2007). Calcium signaling. Cell.

[88] Boitier E., Rea R., Duchen M.R. (1999). Mitochondria exert a negative feedback on the propagation of intracellular Ca2+ waves in rat cortical astrocytes. J Cell Biol.

[89] Tinel H., Cancela J.M., Mogami H., Gerasimenko J.V., Gerasimenko O.V., Tepikin A.V. (1999). Active mitochondria surrounding the pancreatic acinar granule region prevent spreading of inositol trisphosphate-evoked local cytosolic Ca(2+) signals. Embo j.

[90] Babcock D.F., Hille B. (1998). Mitochondrial oversight of cellular Ca2+ signaling. Curr Opin Neurobiol.

[91] Jouaville L.S., Pinton P., Bastianutto C., Rutter G.A., Rizzuto R. (1999). Regulation of mitochondrial ATP synthesis by calcium: evidence for a long-term metabolic priming. Proc Natl Acad Sci U S A.

[92] McCormack J.G., Halestrap A.P., Denton R.M. (1990). Role of calcium ions in regulation of mammalian intramitochondrial metabolism. Physiol Rev.

[93] Wei Y.H., Lu C.Y., Wei C.Y., Ma Y.S., Lee H.C. (2001). Oxidative stress in human aging and mitochondrial disease-consequences of defective mitochondrial respiration and impaired antioxidant enzyme system. Chin J Physiol.

[94] Selivanov V.A., Votyakova T.V., Pivtoraiko V.N., Zeak J., Sukhomlin T., Trucco M. (2011). Reactive oxygen species production by forward and reverse electron fluxes in the mitochondrial respiratory chain. PLoS Comput Biol.

[95] Van Houten B., Woshner V., Santos J.H. (2006). Role of mitochondrial DNA in toxic responses to oxidative stress. DNA Repair (Amst).

[96] Ghezzi D., Zeviani M. (2012). Assembly factors of human mitochondrial respiratory chain complexes: physiology and pathophysiology. Adv Exp Med Biol.

[97] Valko M., Leibfritz D., Moncol J., Cronin M.T., Mazur M., Telser J. (2007). Free radicals and antioxidants in normal physiological functions and human disease. Int J Biochem Cell Biol.

[98] Toescu E.C. (2005). Normal brain ageing: models and mechanisms. Philos Trans R Soc Lond B Biol Sci.

[99] Douarre C., Sourbier C., Dalla Rosa I., Brata Das B., Redon C.E., Zhang H. (2012). Mitochondrial topoisomerase I is critical for mitochondrial integrity and cellular energy metabolism. PLoS One.

[100] Joshi G., Sultana R., Perluigi M., Butterfield D.A. (2005). In vivo protection of synaptosomes from oxidative stress mediated by Fe2+/H2O2 or 2,2-azobis-(2-amidinopropane) dihydrochloride by the glutathione mimetic tricyclodecan-9-yl-xanthogenate. Free Radic Biol Med.

[101] Martin L.J. (2010). The mitochondrial permeability transition pore: a molecular target for amyotrophic lateral sclerosis therapy. Biochim Biophys Acta.

[102] Brand M.D., Affourtit C., Esteves T.C., Green K., Lambert A.J., Miwa S. (2004). Mitochondrial superoxide: production, biological effects, and activation of uncoupling proteins. Free Radic Biol Med.

[103] Liguori I., Russo G., Curcio F., Bulli G., Aran L., Della-Morte D. (2018). Oxidative stress, aging, and diseases. Clin Interv Aging.

[104] Giorgi C., Marchi S., Simoes I.C.M., Ren Z., Morciano G., Perrone M. (2018). Mitochondria and Reactive Oxygen Species in Aging and Age-Related Diseases. Int Rev Cell Mol Biol.

[105] Viña J., Borras C., Abdelaziz K.M., Garcia-Valles R., Gomez-Cabrera M.C. (2013). The free radical theory of aging revisited: the cell signaling disruption theory of aging. Antioxid Redox Signal.

[106] Mittal M., Siddiqui M.R., Tran K., Reddy S.P., Malik A.B. (2014). Reactive oxygen species in inflammation and tissue injury. Antioxid Redox Signal.

[107] Fokam D., Hoskin D. (2020). Instrumental role for reactive oxygen species in the inflammatory response. FBL.

[108] Abramov A.Y., Potapova E.V., Dremin V.V., Dunaev A.V. (2020). Interaction of oxidative stress and misfolded proteins in the mechanism of neurodegeneration. Life (Basel).

[109] Tu B.P., Weissman J.S. (2004). Oxidative protein folding in eukaryotes: mechanisms and consequences. J Cell Biol.

[110] Zhang S., Xin W., Anderson G.J., Li R., Gao L., Chen S. (2022). Double-edge sword roles of iron in driving energy production versus instigating ferroptosis. Cell Death Dis.

[111] Galaris D., Barbouti A., Pantopoulos K. (2019). Iron homeostasis and oxidative stress: An intimate relationship. Biochim Biophys Acta Mol Cell Res.

[112] Kohgo Y., Ikuta K., Ohtake T., Torimoto Y., Kato J. (2008). Body iron metabolism and pathophysiology of iron overload. Int J Hematol.

[113] Mangan D. (2021). Iron: an underrated factor in aging. Aging (Albany NY).

[114] Chen W.J., Kung G.P., Gnana-Prakasam J.P. (2022). Role of iron in aging related diseases. Antioxidants (Basel).

[115] Fairweather-Tait S.J., Wawer A.A., Gillings R., Jennings A., Myint P.K. (2014). Iron status in the elderly. Mech Ageing Dev.

[116] Ko E., Seo H.W., Jung G. (2018). Telomere length and reactive oxygen species levels are positively associated with a high risk of mortality and recurrence in hepatocellular carcinoma. Hepatology.

[117] Kordowitzki P. (2021). Oxidative stress induces telomere dysfunction and shortening in human oocytes of advanced age donors. Cells.

[118] Lin J., Epel E. (2022). Stress and telomere shortening: Insights from cellular mechanisms. Ageing Res Rev.

[119] Barnes R.P., de Rosa M., Thosar S.A., Detwiler A.C., Roginskaya V., Van Houten B. (2022). Telomeric 8-oxo-guanine drives rapid premature senescence in the absence of telomere shortening. Nat Struct Mol Biol.

[120] Fouquerel E., Barnes R.P., Uttam S., Watkins S.C., Bruchez M.P., Opresko P.L. (2019). Targeted and persistent 8-oxoguanine base damage at telomeres promotes telomere loss and crisis. Mol Cell.

[121] Rackham O.J.L., Langley S.R., Oates T., Vradi E., Harmston N., Srivastava P.K. (2017). A Bayesian approach for analysis of whole-genome bisulfite sequencing data identifies disease-associated changes in DNA methylation. Genetics.

[122] Doerfler W., Böhm P. (2006).

[123] Hore T.A., von Meyenn F., Ravichandran M., Bachman M., Ficz G., Oxley D. (2016). Retinol and ascorbate drive erasure of epigenetic memory and enhance reprogramming to naïve pluripotency by complementary mechanisms. Proc Natl Acad Sci.

[124] Bannister A.J., Kouzarides T. (2011). Regulation of chromatin by histone modifications. Cell Res.

[125] Kushwah N., Bora K., Maurya M., Pavlovich M.C., Chen J. (2023). Oxidative stress and antioxidants in age-related macular degeneration. Antioxidants [Internet].

[126] Belarbi K., Cuvelier E., Bonte M.-A., Desplanque M., Gressier B., Devos D. (2020). Glycosphingolipids and neuroinflammation in Parkinson’s disease. Mol Neurodegener.

[127] Moreno-García A., Kun A., Calero O., Medina M., Calero M. (2018). An overview of the role of lipofuscin in age-related neurodegeneration. Front Neurosci.

[128] Katz M.L., Stientjes H.J., Gao C.L., Christianson J.S. (1993). Iron-induced accumulation of lipofuscin-like fluorescent pigment in the retinal pigment epithelium. Invest Ophthalmol Vis Sci.

[129] Ilie O.D., Ciobica A., Riga S., Dhunna N., McKenna J., Mavroudis I. (2020). Mini-review on lipofuscin and aging: focusing on the molecular interface, the biological recycling mechanism, oxidative stress, and the gut-brain axis functionality. Medicina (Kaunas).

[130] Pan C., Banerjee K., Lehmann G.L., Almeida D., Hajjar K.A., Benedicto I. (2021). Lipofuscin causes atypical necroptosis through lysosomal membrane permeabilization. Proc Natl Acad Sci U S A.

[131] Terman A., Brunk U.T. (1998). Lipofuscin: mechanisms of formation and increase with age. Apmis.

[132] Escrevente C., Falcão A.S., Hall M.J., Lopes-da-Silva M., Antas P., Mesquita M.M. (2021). Formation of lipofuscin-like autofluorescent granules in the retinal pigment epithelium requires lysosome dysfunction. Invest Ophthalmol Vis Sci.

[133] Höhn A., Grune T. (2013). Lipofuscin: formation, effects and role of macroautophagy. Redox Biol.

[134] König J., Ott C., Hugo M., Jung T., Bulteau A.L., Grune T. (2017). Mitochondrial contribution to lipofuscin formation. Redox Biol.

[135] Couve E., Osorio R., Schmachtenberg O. (2012). Mitochondrial Autophagy and Lipofuscin Accumulation in Aging Odontoblasts. Journal of Dental Research.

[136] Alzheimer's Association Report (2023). Alzheimer's disease facts and figures. Alzheimer's Dement.

[137] Compagnoni G.M., Fonzo A.D., Corti S., Comi G.P., Bresolin N., Masliah E. (2020). The role of mitochondria in neurodegenerative diseases: the lesson from Alzheimer’s disease and Parkinson’s disease. Mol Neurobiol.

[138] Swerdlow R.H. (2017). Mitochondria and mitochondrial cascades in Alzheimer's disease. J Alzheimer's Dis.

[139] Oliver D.M.A., Reddy P.H. (2019). Molecular basis of Alzheimer's disease: Focus on mitochondria. J Alzheimer's Dis.

[140] Supnet C., Bezprozvanny I. (2010). The dysregulation of intracellular calcium in Alzheimer disease. Cell Calcium.

[141] Wang W., Zhao F., Ma X., Perry G., Zhu X. (2020). Mitochondria dysfunction in the pathogenesis of Alzheimer's disease: recent advances. Mol Neurodegener.

[142] Wang J., Chen G.-J. (2016). Mitochondria as a therapeutic target in Alzheimer's disease. Genes Dis.

[143] Green K.N., Steffan J.S., Martinez-Coria H., Sun X., Schreiber S.S., Thompson L.M. (2008). Nicotinamide restores cognition in Alzheimer's disease transgenic mice via a mechanism involving sirtuin inhibition and selective reduction of Thr231-phosphotau. J Neurosci.

[144] Liu D., Pitta M., Jiang H., Lee J.-H., Zhang G., Chen X. (2013). Nicotinamide forestalls pathology and cognitive decline in Alzheimer mice: evidence for improved neuronal bioenergetics and autophagy procession. Neurobiol Aging.

[145] Chhunchha B., Kubo E., Krueger R.R., Singh D.P. (2023). Hydralazine revives cellular and ocular lens health-span by ameliorating the aging and oxidative-dependent loss of the Nrf2-activated cellular stress response. Antioxidants [Internet].

[146] Maheshwari M., Roberts J.K., DeSutter B., Duong K.T., Tingling J., Fawver J.N. (2010). Hydralazine modifies Aβ fibril formation and prevents modification by lipids in vitro. Biochemistry.

[147] Wright K.M., Bollen M., David J., Speers A.B., Brandes M.S., Gray N.E. (2022). Pharmacokinetics and pharmacodynamics of key components of a standardized centella asiatica product in cognitively impaired older adults: A phase 1, double-blind, randomized clinical trial. Antioxidants [Internet].

[148] Iskusnykh I.Y., Zakharova A.A., Pathak D. (2022). Glutathione in brain disorders and aging. Molecules [Internet].

[149] Kellar D., Craft S. (2020). Brain insulin resistance in Alzheimer's disease and related disorders: mechanisms and therapeutic approaches. Lancet Neurol.

[150] Ailioaie L.M., Ailioaie C., Litscher G. (2023). Photobiomodulation in Alzheimer’s disease—A complementary method to state-of-the-art pharmaceutical formulations and nanomedicine?. Pharmaceutics [Internet].

[151] Enengl J., Hamblin M.R., Dungel P. (2020). Photobiomodulation for Alzheimer’s disease: Translating basic research to clinical application. J Alzheimer's Dis.

[152] Talbott E.O., Malek A.M., Lacomis D., Aminoff M.J., Boller F., Swaab D.F. (2016).

[153] Goutman S.A., Hardiman O., Al-Chalabi A., Chió A., Savelieff M.G., Kiernan M.C. (2022). Emerging insights into the complex genetics and pathophysiology of amyotrophic lateral sclerosis. Lancet Neurol.

[154] Wood A., Gurfinkel Y., Polain N., Lamont W., Lyn Rea S. (2021). Molecular Mechanisms Underlying TDP-43 Pathology in Cellular and Animal Models of ALS and FTLD. Int J Mol Sci [Internet].

[155] Nguyen H.P., Van Broeckhoven C., van der Zee J. (2018). ALS genes in the genomic era and their implications for FTD. Trends Genet.

[156] Renton A.E., Chiò A., Traynor B.J. (2014). State of play in amyotrophic lateral sclerosis genetics. Nat Neurosci.

[157] Wang T., Liu H., Itoh K., Oh S., Zhao L., Murata D. (2021). C9orf72 regulates energy homeostasis by stabilizing mitochondrial complex I assembly. Cell Metab.

[158] Dafinca R., Barbagallo P., Talbot K. (2021). The role of mitochondrial dysfunction and ER stress in TDP-43 and C9ORF72 ALS. Front Cell Neurosci.

[159] (2023). FDA approves treatment of amyotrophic lateral sclerosis associated with a mutation in the SOD1 gene.

[160] Miller T.M., Pestronk A., David W., Rothstein J., Simpson E., Appel S.H. (2013). An antisense oligonucleotide against SOD1 delivered intrathecally for patients with SOD1 familial amyotrophic lateral sclerosis: a phase 1, randomised, first-in-man study. Lancet Neurol.

[161] Liu Y., Andreucci A., Iwamoto N., Yin Y., Yang H., Liu F. (2022). Preclinical evaluation of WVE-004, an investigational stereopure oligonucleotide for the treatment of C9orf72-associated ALS or FTD. Mol Ther Nucl Acids.

[162] (23 May 2023). Wave Life Sciences Announces Topline Results from Phase 1b/2a FOCUS-C9 Study of WVE-004 for C9orf72-associated Amyotrophic Lateral Sclerosis and Frontotemporal Dementia.

[163] Jimenez-Sanchez M., Licitra F., Underwood B.R., Rubinsztein D.C. (2017). Huntington's disease: Mechanisms of pathogenesis and therapeutic strategies. Cold Spring Harb Perspect Med.

[164] Tabrizi S.J., Flower M.D., Ross C.A., Wild E.J. (2020). Huntington disease: new insights into molecular pathogenesis and therapeutic opportunities. Nat Rev Neurol.

[165] Kim J., Moody J.P., Edgerly C.K., Bordiuk O.L., Cormier K., Smith K. (2010). Mitochondrial loss, dysfunction and altered dynamics in Huntington's disease. Hum Mol Genet.

[166] Reddy P.H. (2014). Increased mitochondrial fission and neuronal dysfunction in Huntington's disease: implications for molecular inhibitors of excessive mitochondrial fission. Drug Discov Today.

[167] Liu C., Fu Z., Wu S., Wang X., Zhang S., Chu C. (2022). Mitochondrial HSF1 triggers mitochondrial dysfunction and neurodegeneration in Huntington's disease. EMBO Mol Med.

[168] Exner N., Lutz A.K., Haass C., Winklhofer K.F. (2012). Mitochondrial dysfunction in Parkinson's disease: molecular mechanisms and pathophysiological consequences. Embo j.

[169] Moon H.E., Paek S.H. (2015). Mitochondrial dysfunction in Parkinson's disease. Exp Neurobiol.

[170] Borsche M., Pereira S.L., Klein C., Grunewald A. (2021). Mitochondria and Parkinson's disease: Clinical, molecular, and translational aspects. J Parkinsons Dis.

[171] Hertz N.T., Berthet A., Sos M.L., Thorn K.S., Burlingame A.L., Nakamura K. (2013). A neo-substrate that amplifies catalytic activity of parkinson's-disease-related kinase PINK1. Cell.

[172] Blagov A.V., Goncharov A.G., Babich O.O., Larina V.V., Orekhov A.N., Melnichenko A.A. (2022). Prospects for the development of Pink1 and Parkin activators for the treatment of Parkinson's disease. Pharmaceutics.

[173] Zeuner K.E., Schäffer E., Hopfner F., Brüggemann N., Berg D. (2019). Progress of pharmacological approaches in Parkinson's disease. Clin Pharmacol Ther.

[174] Bender A., Samtleben W., Elstner M., Klopstock T. (2008). Long-term creatine supplementation is safe in aged patients with Parkinson disease. Nutr Res.

[175] Burchell V.S., Gandhi S., Deas E., Wood N.W., Abramov A.Y., Plun-Favreau H. (2010). Targeting mitochondrial dysfunction in neurodegenerative disease: Part I. Expert Opin Ther Targets.

[176] Knopman D.S., Amieva H., Petersen R.C., Chételat G., Holtzman D.M., Hyman B.T. (2021). Alzheimer disease. Nat Rev Dis Prim.

[177] Lin M.T., Beal M.F. (2006). Mitochondrial dysfunction and oxidative stress in neurodegenerative diseases. Nature.

[178] Niedzielska E., Smaga I., Gawlik M., Moniczewski A., Stankowicz P., Pera J. (2016). Oxidative stress in neurodegenerative diseases. Mol Neurobiol.

[179] Pearson A., Ajoy R., Crynen G., Reed J.M., Algamal M., Mullan M. (2020). Molecular abnormalities in autopsied brain tissue from the inferior horn of the lateral ventricles of nonagenarians and Alzheimer disease patients. BMC Neurol.

[180] Puspita L., Chung S.Y., Shim J-w (2017). Oxidative stress and cellular pathologies in Parkinson’s disease. Mol Brain.

[181] Cheignon C., Tomas M., Bonnefont-Rousselot D., Faller P., Hureau C., Collin F. (2018). Oxidative stress and the amyloid beta peptide in Alzheimer's disease. Redox Biol.

[182] Tönnies E., Trushina E. (2017). Oxidative stress, synaptic dysfunction, and Alzheimer's disease. J Alzheimers Dis.

[183] Murphy M.P., Hartley R.C. (2018). Mitochondria as a therapeutic target for common pathologies. Nat Rev Drug Discov.

[184] Magalhaes J., Tresse E., Ejlerskov P., Hu E., Liu Y., Marin A. (2021). PIAS2-mediated blockade of IFN-β signaling: a basis for sporadic Parkinson disease dementia. Mol Psychiatry.

[185] George M., Tharakan M., Culberson J., Reddy A.P., Reddy P.H. (2022). Role of Nrf2 in aging, Alzheimer's and other neurodegenerative diseases. Ageing Res Rev.

[186] Kahroba H., Ramezani B., Maadi H., Sadeghi M.R., Jaberie H., Ramezani F. (2021). The role of Nrf2 in neural stem/progenitors cells: From maintaining stemness and self-renewal to promoting differentiation capability and facilitating therapeutic application in neurodegenerative disease. Ageing Res Rev.

[187] Kim S., Indu Viswanath A.N., Park J.H., Lee H.E., Park A.Y., Choi J.W. (2020). Nrf2 activator via interference of Nrf2-Keap1 interaction has antioxidant and anti-inflammatory properties in Parkinson's disease animal model. Neuropharmacology.

[188] Niu Y., Zhang J., Dong M. (2021). Nrf2 as a potential target for Parkinson's disease therapy. J Mol Med (Berl).

[189] Buendia I., Michalska P., Navarro E., Gameiro I., Egea J., León R. (2016). Nrf2-ARE pathway: An emerging target against oxidative stress and neuroinflammation in neurodegenerative diseases. Pharmacol Ther.

[190] Rivetti di Val Cervo P., Besusso D., Conforti P., Cattaneo E. (2021). hiPSCs for predictive modelling of neurodegenerative diseases: dreaming the possible. Nat Rev Neurol.

